# Rapid, point-of-care diagnosis of tuberculosis with novel Truenat assay: Cost-effectiveness analysis for India’s public sector

**DOI:** 10.1371/journal.pone.0218890

**Published:** 2019-07-02

**Authors:** David J. Lee, Nagalingeswaran Kumarasamy, Stephen C. Resch, Gomathi N. Sivaramakrishnan, Kenneth H. Mayer, Srikanth Tripathy, A. David Paltiel, Kenneth A. Freedberg, Krishna P. Reddy

**Affiliations:** 1 Harvard Medical School, Boston, Massachusetts, United States of America; 2 Medical Practice Evaluation Center, Massachusetts General Hospital, Boston, Massachusetts, United States of America; 3 Chennai Antiviral Research and Treatment Clinical Research Site, Voluntary Health Services, Chennai, India; 4 Center for Health Decision Science, Harvard T.H. Chan School of Public Health, Boston, Massachusetts, United States of America; 5 Department of Health Policy and Management, Harvard T.H. Chan School of Public Health, Boston, Massachusetts, United States of America; 6 National Institute for Research in Tuberculosis, Chennai, India; 7 The Fenway Institute, Fenway Health, Boston, Massachusetts, United States of America; 8 Beth Israel Deaconess Medical Center, Boston, Massachusetts, United States of America; 9 Yale School of Public Health, New Haven, Connecticut, United States of America; 10 Division of General Internal Medicine, Massachusetts General Hospital, Boston, Massachusetts, United States of America; 11 Division of Infectious Diseases, Massachusetts General Hospital, Boston, Massachusetts, United States of America; 12 Division of Pulmonary and Critical Care Medicine, Massachusetts General Hospital, Boston, Massachusetts, United States of America; Rutgers Biomedical and Health Sciences, UNITED STATES

## Abstract

**Background:**

Truenat is a novel molecular assay that rapidly detects tuberculosis (TB) and rifampicin-resistance. Due to the portability of its battery-powered testing platform, it may be valuable in peripheral healthcare settings in India.

**Methods:**

Using a microsimulation model, we compared four TB diagnostic strategies for HIV-negative adults with presumptive TB: (1) sputum smear microscopy in designated microscopy centers (DMCs) (*SSM*); (2) Xpert MTB/RIF in DMCs (*Xpert*); (3) Truenat in DMCs (*Truenat DMC*); and (4) Truenat for point-of-care testing in primary healthcare facilities (*Truenat POC*). We projected life expectancy, costs, incremental cost-effectiveness ratios (ICERs), and 5-year budget impact of deploying *Truenat POC* in India’s public sector. We defined a strategy “cost-effective” if its ICER was <US$990/year-of-life saved (YLS). Model inputs included: TB prevalence, 15% (among those not previously treated for TB) and 27% (among those previously treated for TB); sensitivity for TB detection, 89% (Xpert) and 86% (Truenat); per test cost, $12.63 (Xpert) and $13.20 (Truenat); and linkage-to-care after diagnosis, 84% (DMC) and 95% (POC). We varied these parameters in sensitivity analyses.

**Results:**

Compared to *SSM*, *Truenat POC* increased life expectancy by 0.39 years and was cost-effective (ICER $210/YLS). Compared to *Xpert*, *Truenat POC* increased life expectancy by 0.08 years due to improved linkage-to-care and was cost-effective (ICER $120/YLS). In sensitivity analysis, the cost-effectiveness of *Truenat POC*, relative to *Xpert*, depended on the diagnostic sensitivity of Truenat and linkage-to-care with Truenat. Deploying *Truenat POC* instead of *Xpert* increased 5-year expenditures by $270 million, due mostly to treatment costs. Limitations of our study include uncertainty in Truenat’s sensitivity for TB and not accounting for the “start-up” costs of implementing Truenat in the field.

**Conclusions:**

Used at the point-of-care in India, Truenat for TB diagnosis should improve linkage-to-care, increase life expectancy, and be cost-effective compared with smear microscopy or Xpert.

## Introduction

With approximately 2.8 million cases annually, India has the world’s highest incidence of tuberculosis (TB) [1]. However, due to the widespread use of diagnostics with low sensitivity (e.g., sputum smear microscopy) and low linkage-to-care rates, over 25% of patients seeking care in India’s public sector are neither diagnosed nor started on treatment [2].

New rapid molecular diagnostics could dramatically increase TB detection and linkage-to-care, which are key components of both the World Health Organization’s (WHO) End TB Strategy and India’s National Strategic Plan for Tuberculosis Elimination 2017–2025 [1,3]. The Xpert MTB/RIF assay (Cepheid, Sunnyvale, CA, USA) is currently the only WHO-endorsed molecular test able to rapidly detect both TB and rifampicin (RIF)-resistance [1]. While there has been interest in deploying Xpert in peripheral laboratories [3–6], its decentralization may be limited by infrastructure requirements, including continuous power supply and air-conditioning [7,8].

Truenat (Molbio Diagnostics/Bigtec Labs, Goa/Bengaluru, India), is a new chip-based, micro real-time polymerase chain reaction (PCR) test that detects tubercle bacilli in sputum samples in approximately one hour [1,9,10]. Upon receiving a positive test result, an “add-on” chip can be used to detect RIF-resistance, adding another one hour of test time. The test is prepared and run on the battery-powered Truelab system, which includes the sample preparation device (i.e., machine for DNA extraction and purification from the sputum sample) and the PCR analyzer device, available in 1-, 2-, or 4-module configurations with the lattermost capable of testing four samples simultaneously. Due to the portability of this testing platform, Truenat may be valuable in peripheral healthcare settings, such as designated microscopy centers (DMCs) and primary healthcare facilities in India.

If used as a point-of-care (POC) test within primary healthcare facilities, Truenat could increase treatment initiation by reducing turnaround time for test results and decreasing the need for laboratory referrals [2,11]. However, uncertainties in parameter values, such as test characteristics and linkage-to-care, must be investigated. In resource-constrained settings, the potential benefits of Truenat must also be weighed against its costs. Using a mathematical model, therefore, we projected the clinical impact, costs, and cost-effectiveness of Truenat, as a replacement for smear microscopy or Xpert. We also evaluated the budget impact of deploying Truenat widely in India’s public sector.

## Methods

### Analytic overview

We expanded the Cost-Effectiveness of Preventing AIDS Complications-International (CEPAC-I) model, a validated and widely published, individual-based Monte Carlo state-transition model, developed and written in C++ [12–14], to account for TB natural history, diagnosis, and treatment. We simulated a cohort of adult, HIV-negative patients with presumptive pulmonary TB, defined as individuals aged ≥15 years with cough of ≥2 weeks duration [4,15], undergoing TB testing at DMCs and their attached primary healthcare facilities (e.g., primary health centers, hospital outpatient clinics, etc.) under India’s Revised National Tuberculosis Control Programme (RNTCP). Although HIV is an important risk factor for TB, we focused on the HIV-negative population, among whom 97% of new TB cases in India occur [1].

We compared the clinical and economic outcomes of four TB diagnostic strategies: (1) sputum smear microscopy in DMCs (*SSM*); (2) Xpert in DMCs (*Xpert*); (3) Truenat in DMCs (*Truenat DMC*); and (4) Truenat for point-of-care testing in primary healthcare facilities (*Truenat POC*). In each strategy, patients for whom clinical suspicion of TB remains high despite a negative test result may undergo additional testing. Patients previously treated for TB, and therefore at higher risk of drug-resistance, may receive additional tests to confirm multidrug-resistant TB (MDR-TB) (S1 Appendix; Figures A-C in S1 Appendix).

Model-generated outcomes included the correct detection and linkage-to-care of TB cases; life expectancy; lifetime TB-related healthcare costs; and incremental cost-effectiveness ratio (ICER), the difference between two strategies in costs (2017 US dollars) divided by the difference in life-years. We considered a strategy cost-effective if its ICER was less than US$990/year-of-life saved (YLS), an opportunity-based cost-effectiveness threshold that is 50% of India’s 2017 gross domestic product (GDP) *per capita* (S1 Appendix). We projected clinical and economic outcomes over patients’ lifetimes, while also considering shorter time horizons. We report outcomes discounted 3%/year for cost-effectiveness analysis and undiscounted outcomes for clinical and budget evaluations [16].

### Model overview

A simulated cohort of patients enters the model upon seeking care in India’s public sector for symptoms suggestive of TB. They undergo a TB diagnostic protocol per national guidelines [4,15]. The model draws randomly from user-defined characteristics (e.g., age, sex, TB status), informed by a recent implementation study of Xpert in India for patients with presumptive TB [4]. As individuals transition monthly through “states” of TB progression and treatment, the model tracks clinical outcomes (e.g., cure, relapse, life-years accrued) and monthly TB-related healthcare costs (e.g., diagnostic tests, drugs, clinic visits). Throughout the simulation, all individuals are subject to age- and sex-stratified background mortality risks specific for India, while those with active, untreated TB have an excess mortality risk. Model details are in the S1 Appendix and at http://www.massgeneral.org/mpec/cepac/.

To initiate TB treatment, a simulated individual must: (1) complete the diagnostic pathway, including retrieving test results; (2) receive a diagnosis of TB and/or drug-resistance, as determined by test characteristics; and (3) link to a primary healthcare facility that will initiate and monitor TB treatment (“linkage-to-care”). In the diagnostic pathway for *Truenat DMC* or *Truenat POC*, an individual undergoes testing for RIF-resistance only if the individual first receives a positive test result for TB. Those who receive a TB diagnosis via *Truenat POC* have a higher probability of linking to care for TB treatment compared to those who are diagnosed in DMCs, who require a referral to a primary healthcare facility (S1 Appendix) [2].

### Base case input parameters

#### Cohort characteristics and TB prevalence

Cohort characteristics were derived from an implementation study of Xpert for individuals with presumptive TB in DMCs of 18 sub-district level TB program units, chosen for being geographically and demographically representative of the national population in India (Table 1) [4]. Mean age was 41 years, 36% were women, and 17% had been previously treated for TB. Among patients with presumptive TB, we assumed TB prevalence was 15% for patients with no prior TB treatment and 27% for previously treated patients (S1 Appendix) [4].

**Table 1:** Input parameters for model-based analysis of TB diagnostic strategies for individuals with presumptive TB in India.

Parameter	Base case	Range^a^	References
**Baseline cohort characteristics**			
Age, years, mean (SD)	41.4 (16.1)	—	[4]
Men/Women	64/36%	—	[4]
Proportion previously treated for TB	17%	7–27%	[4]
Prevalence of TB			
among those not previously treated for TB	15%	8–23%	S1 Appendix
among those previously treated for TB	27%	18–40%	S1 Appendix
Prevalence of MDR-TB			
among those not previously treated for TB	6%	4–7%	[17]
among those previously treated for TB	36%	29–42%	[17]
**Diagnostic tests**			
Sputum smear microscopy			
Sensitivity (2 samples)	64%	60–69%	[18]
Specificity (2 samples)	98%	97–99%	[18]
Proportion of patients who provide second sputum sample	89%	85–93%	[2]
Cost per test (USD 2017)	$0.86	$0.24–1.58	[19]
Clinical diagnosis for smear-negative patients^b^			
Sensitivity	16%	6–26%	[20]
Specificity	94%	84–100%	[20]
Proportion of smear-negative patients who undergo a clinical diagnostic work-up	39%	20–39%	[2]
Cost per patient (USD 2017)	$8.24	$7.17–9.28	[21]
Xpert			
Sensitivity, TB detection	89%	85–92%	[22]
Specificity, TB detection	99%	98–99%	[22]
Sensitivity, RIF-resistance detection	95%	90–97%	[22]
Specificity, RIF-resistance detection	98%	97–99%	[22]
Probability of test failure (for power or temperature issue)	1%	0–5%	[6]
Cost per test (USD 2017)	$12.63	$11.47 –$14.84	[19]
Truenat			
Sensitivity, TB detection	86%	66–100%	Methods; [9]
Specificity, TB detection	99%	80–100%	Methods
Sensitivity, RIF-resistance detection^c^	94%	74–100%	S1 Appendix
Specificity, RIF-resistance detection^c^	98%	88–100%	S1 Appendix
Cost per test (USD 2017)	$13.20	$12.75 –$13.79	Communication with manufacturer; [19]
Liquid culture & DST			
Culture sensitivity, TB detection	100%	—	Gold standard assumption
Culture specificity, TB detection	100%	—	
DST sensitivity, MDR-TB detection	100%	—	Gold standard assumption
DST specificity, MDR-TB detection	100%	—	
Cost per test, liquid culture (USD 2017)^d^	$13.30	$10.32 –$16.29	[19]
Cost per test, DST (USD 2017)^d^	$30.93	$27.23 –$34.63	[19]
**Treatment of TB**			
Linkage-to-care			
after DMC-based test	84%	80–88%	S1 Appendix
after POC test (i.e., *Truenat POC*)	95%	88–100%	S1 Appendix
Monthly probability of loss to follow-up during treatment^e^	1%	0.008–2%^f^	[5]
Monthly cost of treatment^g^			
First-line regimen, 6 months (USD 2017)	$28.13	$24.13 –$32.49	[19,23,24]
Retreatment regimen, 8 months (USD 2017)	$32.25	$28.30 –$36.23	[19,23,24]
Second-line regimen, 24 months (USD 2017)	$104.23	$96.15 –$112.13	[19,23,24]

TB: tuberculosis. MDR-TB: multidrug-resistant tuberculosis. RIF: rifampicin. SD: standard deviation. USD: 2017 United States dollars. C&DST: culture and drug-susceptibility testing. DST: drug-susceptibility testing. DMC: designated microscopy center. POC: point-of-care.

^a^Range used for univariate sensitivity analysis.

^b^Clinical diagnosis includes chest radiography and antibiotic trial.

^c^Sensitivity and specificity of Truenat for RIF-resistance detection are based on the line probe assay as the gold standard (S1 Appendix).

^d^Costs for liquid culture and DST are based on the BACTEC MGIT (BD, Sparks, MD, USA) system [19].

^e^Monthly probability of loss to follow-up is the weighted probability of loss to follow-up during all treatment regimens [5].

^f^Range based on variation across sites.

^g^Treatment costs include drugs, monitoring tests, clinic visits, and hospitalizations (S1 Appendix).

#### Diagnostic tests

Sensitivity and specificity of smear microscopy for two sputum samples were 64% and 98% compared to culture (Table 1) [18]. Sensitivity and specificity of Xpert were 89% and 99% compared to culture, per the results of a meta-analysis [22]. One validation study in India reports Truenat’s sensitivity for TB detection as 96% compared to Xpert [9]. To compare Truenat to culture, we multiplied 96% (Truenat’s sensitivity against Xpert) by 89% (Xpert’s sensitivity against culture). Therefore, the sensitivity of Truenat was 86% compared to culture. We assumed 99% specificity for Truenat, similar to Xpert (Table 1) [22]. We varied Truenat sensitivity and specificity in sensitivity analyses.

#### Linkage-to-care and treatment

Linkage-to-care was 84% for patients diagnosed in DMCs and 95% for those diagnosed by a POC test (*Truenat POC* only) [2]. The latter was an evidence-supported assumption (S1 Appendix) that we varied in sensitivity analyses. For patients who received a negative result after POC testing with Truenat but were subsequently diagnosed via culture, linkage-to-care remained 84%. Treatment-related parameters, including loss to follow-up (LTFU), were derived from Indian TB surveillance data (Table 1) [5]. The monthly probability of LTFU remained 1% regardless of the severity of TB-related symptoms. We varied this assumption in sensitivity analysis.

#### Costs

Using a microcosting approach, we derived unit costs of TB care from a health system perspective [19,21,23,24]. We multiplied unit costs by their expected quantities, either as indicated by published guidelines (e.g., number of clinic visits) or as reported in epidemiological studies (e.g., hospitalization rates) (S1 Appendix). The costs per test for sputum smear microscopy, Xpert, and Truenat were $0.86, $12.63, and $13.20 (Table 1), which included costs of overhead and building space, labor, reagents (e.g., cartridges for Xpert, chips for Truenat), and equipment (test instruments). The monthly costs of TB treatment were $28.13 (first-line), $32.25 (retreatment), and $104.23 (second-line), which included the costs of drugs, monitoring tests, clinic visits, and hospitalizations during treatment (S1 Appendix) [19,21,23,24].

We obtained price estimates for the Truenat platform, including the Truenat chip and the Truelab system, from the manufacturer (Sriram Natarajan, Director and CEO of Molbio, personal communication). The price of the Truelab system, which includes the sample preparation device and the 4-module PCR analyzer device (capable of testing four specimens simultaneously), is $14,150. We used this cost in the base case for comparison with the 4-module Xpert system (assumed to be the standard in India). This cost was annualized over the expected lifespan of the Truelab system, discounted 3%/year, and divided by the expected number of tests it would perform annually. The price of the Truenat chip for TB detection is $12.40, and the chip for RIF-resistance detection will be provided free of cost based on an average estimated TB-positive proportion of 20%. These initial price estimates for the public sector may change based on volume commitment by the government. We assumed that overhead, building space, and labor-related costs for Truenat strategies would be similar to those of Xpert [19].

Additional input parameters regarding TB prevalence, natural history, diagnostics, and treatment are in S1 Appendix. The majority of the data inputs in this study, including those related to population demographics, gaps in the TB cascade of care, and diagnostic test and treatment costs, specifically apply to the public sector in India.

### Sensitivity and scenario analyses

To account for uncertainty, we varied key parameters (e.g., test sensitivity, linkage-to-care, costs) across a wide range of possible values, informed by literature whenever possible (Table 1). We evaluated the effect of empirical treatment on cost-effectiveness. Specifically, we considered the scenario in which 16% of those with a negative smear, Xpert, or Truenat result receive empirical treatment (S1 Appendix). We performed a scenario analysis in which patients who received a TB diagnosis under the *Truenat POC* strategy are twice as likely to be lost to follow-up during treatment compared to patients diagnosed under the *SSM* strategy (2% from 1%). This was intended to reflect that patients who are rapidly diagnosed and linked to care with POC testing are likely to have TB symptoms that are less severe and are, therefore, more likely to discontinue TB treatment.

We also evaluated scenarios in which the per-test costs of operating the Truenat platform are higher in primary healthcare facilities than they would be in DMCs due to economies of scale. This was intended to reflect that compared to DMCs, primary healthcare facilities are likely to experience lower test volumes, resulting in the less efficient use of certain material and human resources, such as overhead, building space, equipment (test instruments), and some elements of labor (S1 Appendix). Specifically, we considered the scenarios in which primary healthcare facilities have test volumes 5- and 10-fold lower than those of DMCs, increasing the per-test cost of *Truenat POC* from $13.20 (base case) to $15.32 and $17.96, respectively (S1 Appendix).

We conducted a two-way sensitivity analysis, simultaneously varying Truenat’s sensitivity for TB detection and linkage-to-care at a 5-year horizon, to define the combination of parameter values by which *Truenat POC* would be more economically efficient than *Xpert*. Because the public sector cost of Truenat may decrease based on volume commitment by the Indian government, we conducted this same analysis for a scenario in which the Truenat chip cost is negotiated to 60% of the current estimate (S1 Appendix).

### Budget impact analysis

We projected costs associated with widespread deployment of diagnostic strategies in India’s public sector over a 2-year and 5-year period. We assumed 7.9 million adults with presumptive TB would be tested annually (S1 Appendix).

## Results

### Base case clinical outcomes

*Truenat DMC*, compared to *SSM*, increased life expectancy by 0.30 years (undiscounted) but, compared to *Xpert*, decreased life expectancy by 0.01 years (Table 2). *Truenat POC* was the most effective strategy, increasing life expectancy by 0.39 years compared to *SSM* and by 0.08 years compared to *Xpert*. Compared to *SSM* and *Xpert*, *Truenat POC* also increased the number of TB cases correctly detected and linked to care by 590 and 140, respectively, per 10,000 individuals with presumptive TB.

**Table 2:** Model-generated clinical and economic outcomes of TB diagnostic strategies.

	Cases detected^a^	Cases detected and linked^b^	Lifetime outcomes (per person)				
	per 10,000 individuals with presumptive TB		Life-years^c^		Costs (2017 USD)^d^		ICER ($/YLS)^g^
Strategy			Undisc.	Disc. (3%/y)	Undisc.	Disc. (3%/y)	
***SSM***	1,000	840	31.17	18.58	80	80	–
***Truenat DMC***	1,510	1,270	31.47	18.76	130	120	dominated^h^
***Xpert***	1,530	1,290	31.48	18.76	130^e^	120^e^	dominated^h^
***Truenat POC***	1,510	1,430	31.56	18.80	140	120^f^	210

TB: tuberculosis. SSM: sputum smear microscopy. DMC: designated microscopy center. POC: point-of-care. Undisc: undiscounted. Disc. (3%/y): discounted 3%/year. USD: United States dollars.

ICER: incremental cost-effectiveness ratio. YLS: year-of-life saved.

^a^Number of individuals with presumptive TB seeking care at model entry who were correctly identified as having TB by each strategy.

^b^Number of individuals with presumptive TB seeking care at model entry who were correctly identified as having TB and linked to treatment by each strategy.

^c^Average total number of life-years that is accrued (i.e., the remaining life expectancy) from when an individual enters the model until his/her death, under each strategy.

^d^Average total costs of all TB-related services (e.g., diagnostic tests and TB treatment) that are accrued throughout the patient’s lifetime, under each strategy.

^e^Lifetime cost of *Xpert* is higher than that of *Truenat DMC*, but appears the same due to rounding.

^f^Lifetime cost of *Truenat POC* is higher than that of *Xpert*, but appears the same due to rounding.

^g^ICERs were calculated using exact numbers, then rounded to the nearest $10.

^h^”dominated”: weakly dominated (higher ICER than that of a strategy offering more life-years).

### Base case lifetime costs and cost-effectiveness

Compared to *SSM*, *Truenat DMC* and *Truenat POC* strategies both increased discounted per-patient lifetime costs by ~$40 (Table 2). Compared to *Xpert*, *Truenat DMC* decreased discounted per-patient lifetime costs by $1 and *Truenat POC* increased costs by $5.

While *Truenat DMC* was cost-effective compared to *SSM* (ICER $240/YLS), it resulted in lower life expectancy and higher ICER than *Xpert* and was, therefore, “weakly dominated” (i.e., economically inefficient). *Truenat POC* was cost-effective compared to both *SSM* (ICER $210/YLS) and *Xpert* (ICER $120/YLS). When viewed over different time horizons, *Truenat POC* became cost-effective compared to *Xpert* and *SSM* after 4 and 6 years, and *Xpert* became cost-effective compared to *SSM* after 6 years (Figure E in S1 Appendix). The respective ICERs continued to decrease beyond these time horizons.

### One-way sensitivity and scenario analyses

**Fig 1:**
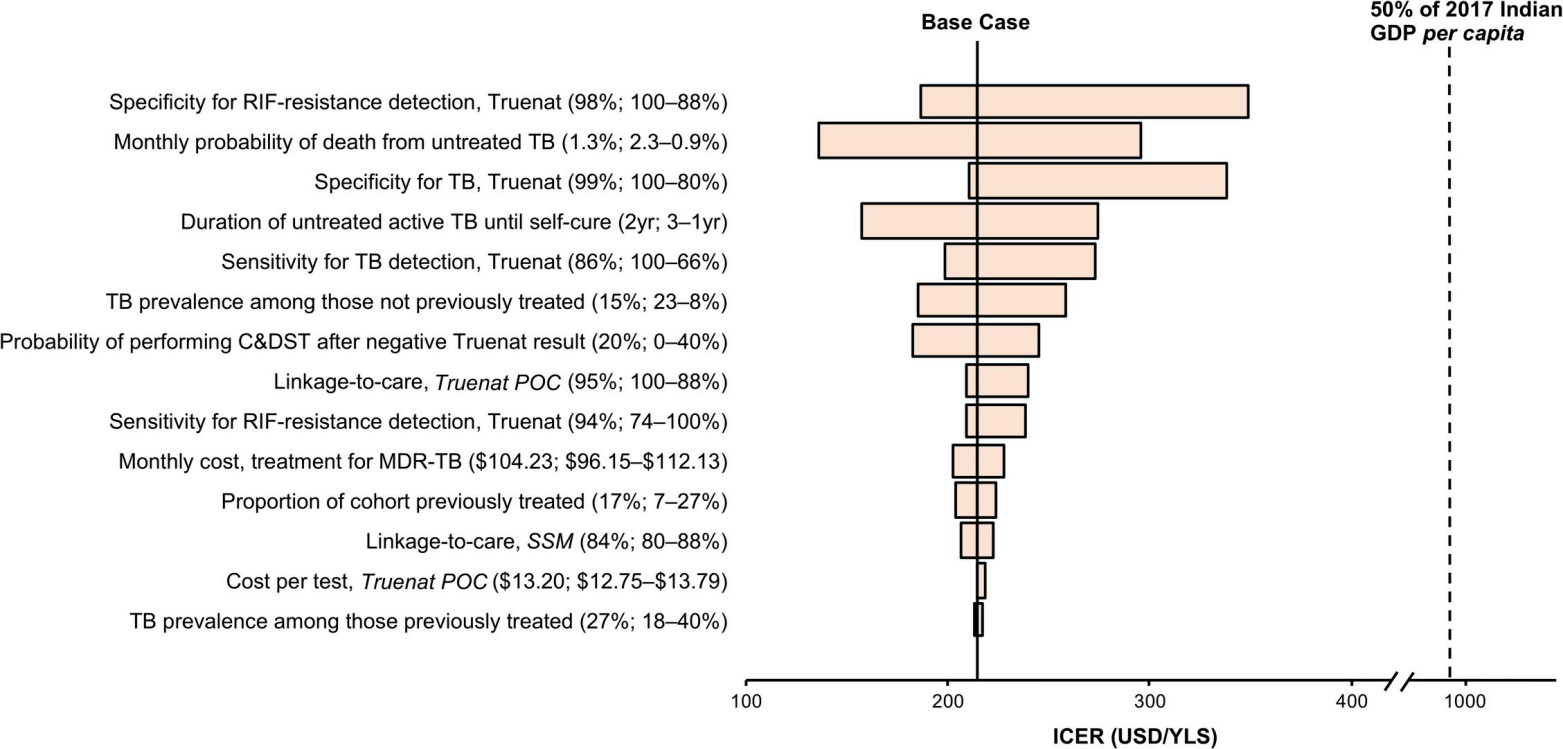
One-way sensitivity analyses of key model parameters, comparing *Truenat POC* to *SSM*, at lifetime horizon. TB: tuberculosis. MDR-TB: multidrug-resistant tuberculosis. RIF: rifampicin. C&DST: culture and drug-susceptibility test. yr: year. POC: point-of-care. “previously treated”: previously treated for TB. GDP: gross domestic product. ICER: incremental cost-effectiveness ratio. USD: United States dollars. YLS: year-of-life saved. Horizontal bars represent ranges of ICERs when varying each model parameter across its plausible range. The vertical dashed line represents 50% of the GDP *per capita* of India in 2017 ($990), which we consider the cost-effectiveness threshold (see Methods). ICERs less than $990/YLS (left of dashed line) are considered cost-effective.

In one-way sensitivity analyses comparing *Truenat POC* to *SSM* at a lifetime horizon, *Truenat POC* remained cost-effective compared to *SSM* across all parameter values analyzed (Fig 1; Figure F in S1 Appendix). Decreasing Truenat’s sensitivity for TB detection by an absolute 20% (from 86% to 66%) resulted in ~27% change in the base case ICER of *Truenat POC* versus *SSM*, at a lifetime horizon. Varying TB and MDR-TB prevalence had little influence on the ICER of *Truenat POC*. Varying the specificity of Truenat across a range of values (80% to 100%) also had little influence on the ICER of *Truenat POC*. While Truenat’s specificity for RIF-resistance detection was the most influential among the parameters considered, the ICER ($350/YLS) that resulted from decreasing the specificity by 10% remained well below the cost-effectiveness threshold of $990/YLS.

When accounting for empirical treatment in 16% of those with a negative smear, Xpert, or Truenat result, *Truenat POC* remained cost-effective compared to *SSM* (ICER $290/YLS) and compared to *Xpert* (ICER $200/YLS). Increasing the monthly probability of LTFU for patients who received a TB diagnosis under the *Truenat POC* strategy to twice the monthly probability of LTFU for patients diagnosed under the *SSM* strategy (2% from 1%) resulted in a minimal change (~3%) in the base case ICER of *Truenat POC* versus *SSM*. In the scenario that primary healthcare facilities have test volumes 5-fold lower than those of DMCs (increasing the per-test cost of *Truenat POC* to $15.32), *Truenat POC* remained cost-effective compared to *SSM* (ICER $220/YLS) and compared to *Xpert* (ICER $170/YLS). In the scenario that primary healthcare facilities have test volumes 10-fold lower than those of DMCs (increasing the per-test cost of *Truenat POC* to $17.96), *Truenat POC* remained cost-effective compared to *SSM* (ICER $240/YLS) and compared to *Xpert* ($240/YLS).

**Fig 2:**
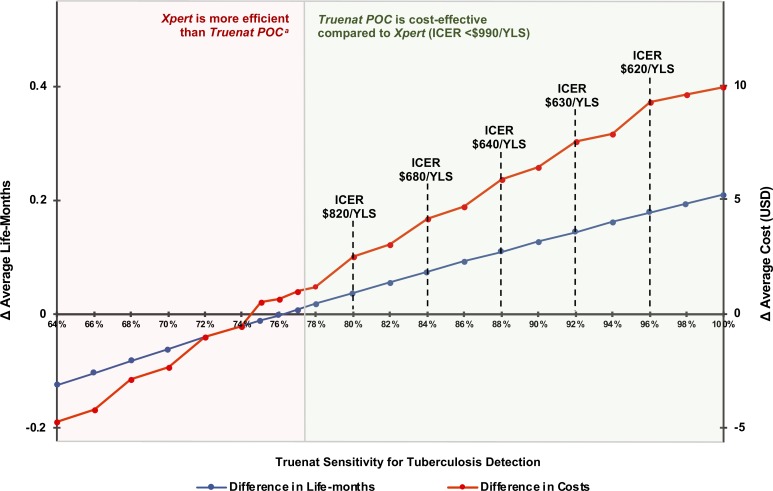
Sensitivity analysis of Truenat sensitivity for TB, comparing *Truenat POC* to *Xpert* at 5-year horizon. TB: tuberculosis. POC: point-of-care. ICER: incremental cost-effectiveness ratio. YLS: year-of-life saved. USD: United States dollars. This plot shows the differences in life expectancy and costs between *Truenat POC* and *Xpert* at a 5-year horizon when varying the sensitivity of Truenat for TB detection. The horizontal axis is the sensitivity of Truenat for TB detection. The blue line corresponds to the left vertical axis, which is the difference in life expectancy between *Truenat POC* and *Xpert*. The red line corresponds to the right vertical axis, which is the difference in per-person lifetime costs between *Truenat POC* and *Xpert*. The ICER (i.e., the difference in costs divided by the difference in life expectancy) is provided at regular intervals of test sensitivity values. For integer values of test sensitivity ≥78% (green panel), *Truenat POC* is cost-effective compared to *Xpert* (ICER <$990/YLS). For integer values *<*78% (red panel), *Xpert* is more efficient than *Truenat POC*. ^a^“*Xpert* is more efficient than *Truenat POC*”: For Truenat sensitivity values <75%, *Xpert* was cost-effective compared to *Truenat POC* (ICER <$990/YLS). At Truenat sensitivity of 75–76%, *Xpert* was cost-saving (lower cost, higher clinical benefit [more life-years accrued]) compared to *Truenat POC*. At Truenat sensitivity of 77%, *Xpert* was decrementally cost-effective (lower cost and lower clinical benefit but with ICER >$990/year-of-life lost [YLL]—that is, at least $990 saved per year-of-life-lost) compared to *Truenat POC*.

We compared *Truenat POC* to *Xpert* over a shorter 5-year horizon, varying Truenat’s sensitivity for TB detection (Fig 2). When Truenat’s sensitivity was ≥78%, *Truenat POC* increased life-years, increased costs, and was cost-effective compared to *Xpert*. The higher cost was driven mostly by the increased number of patients initiating treatment; however, these costs were offset by improvements in clinical outcomes, resulting in overall decreasing ICERs as sensitivity increased. When Truenat’s sensitivity was <75%, *Truenat POC*, compared to *Xpert*, resulted in fewer life-years and lower cost. In this scenario, the ICER of *Xpert* compared to *Truenat POC* was below the cost-effectiveness threshold, indicating that *Xpert* was cost-effective.

### Two-way sensitivity and scenario analyses

**Fig 3:**
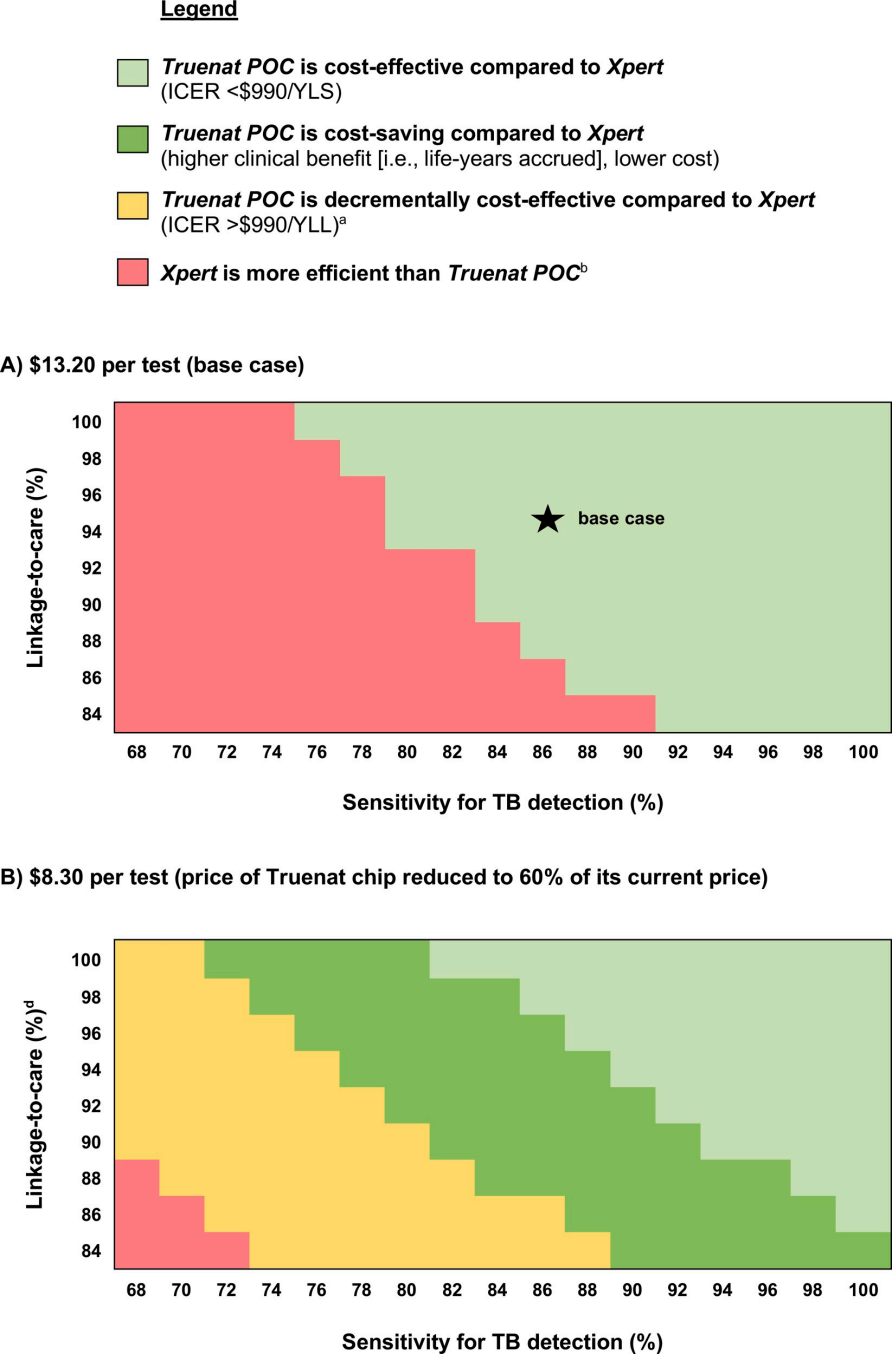
Two-way sensitivity and scenario analysis heat maps, comparing *Truenat POC* to *Xpert* at 5-year horizon. TB: tuberculosis. POC: point-of-care. YLS: year-of-life saved. YLL: year-of- lost. These heat maps evaluate the incremental cost-effectiveness ratio of *Truenat POC* strategy relative to *Xpert* at a 5-year time horizon for different values of Truenat sensitivity for TB detection and linkage-to-care. Each panel displays different costs of Truenat, including the scenario (B), in which the price of the Truenat chip is negotiated to 60% of its current estimate for the public sector (S1 Appendix). Sensitivity of Truenat for TB detection increases from left to right on the horizontal axes. The probability of linking to care upon receiving a positive TB test result with Truenat increases up the vertical axes. ^a^“Decrementally cost-effective”: *Truenat POC* results in lower cost and lower clinical benefit compared to *Xpert*, but with ICER >$990/year-of-life lost (YLL)—that is, at least $990 is saved per year-of-life lost. ^b^“*Xpert* is more efficient than *Truenat POC*”: *Xpert* is either cost-effective (ICER <$990/YLS), cost-saving (lower cost, higher clinical benefit [more life-years accrued]), or decrementally cost-effective (ICER >$990/YLL), compared to *Truenat POC*.

We simultaneously varied Truenat’s sensitivity for TB detection (68–100%) and linkage-to-care (84–100%), comparing *Truenat POC* to *Xpert* over a 5-year horizon (Fig 3A). We kept Xpert’s sensitivity and linkage-to-care at base case values. *Truenat POC*, at 86% sensitivity, was cost-effective when linkage-to-care was ≥88%. This linkage threshold for cost-effectiveness increased as Truenat sensitivity decreased. For sensitivity values ≤74%, *Truenat POC* was not cost-effective at any linkage value. Above 90% sensitivity, *Truenat POC* was cost-effective at linkage values as low as 84% (same linkage as *Xpert*).

In the scenario that Truenat’s chip cost is reduced to 60% of its current estimate, the range of parameter values for which *Truenat POC* was cost-effective (or cost-saving) compared to *Xpert* broadened (Fig 3B). At 95% linkage (as assumed for POC test), *Truenat POC* was cost-effective when sensitivity was ≥88%, cost-saving when sensitivity was 77–87%, and decrementally cost-effective (“much less costly and almost as good”) when sensitivity was ≤76%. When linkage was 84% (typical of DMC), *Truenat POC* was cost-saving when sensitivity was >88% and decrementally cost-effective when sensitivity was 74–88%.

### Budget impact analysis

**Fig 4:**
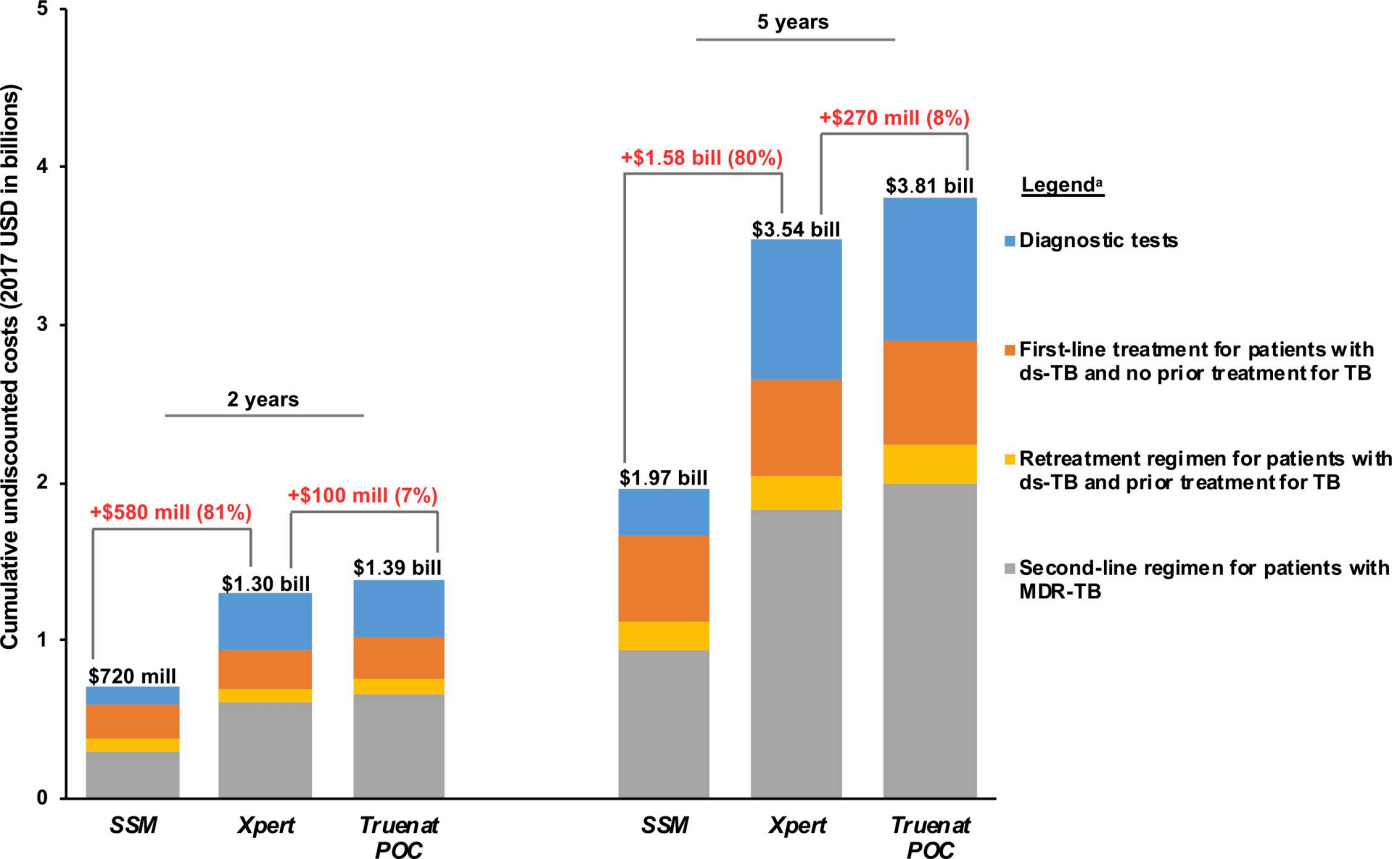
Budget impact analysis over 2 and 5 years. TB: tuberculosis. ds-TB: drug-susceptible tuberculosis. MDR-TB: multidrug-resistant tuberculosis. SSM: sputum smear microscopy. POC: point-of-care. mill: million. bill: billion. Budget impact analysis of full public sector implementation of sputum smear microscopy (*SSM*), *Xpert*, and *Truenat POC* strategies over 2- and 5-year time horizons. Cumulative TB-related costs (2017 USD, billions) are on the vertical axis. This analysis assumes that 7.9 million adults in India are tested each year for symptoms suggestive of TB (S1 Appendix) [4]. All calculations were made using exact numbers before rounding to the nearest $10 million (for costs) and 1% (for percentages). ^a^Each treatment regimen is associated with a frequency of clinic visits and rate of hospitalization during the course of TB treatment, as reported by published guidelines and/or epidemiological data (S1 Appendix). These clinical costs are incorporated into the budget impact projection for each category.

Compared to country-wide use of *SSM* in India’s public sector, scaling up *Xpert* increased cumulative TB-related healthcare expenditures by $580 million (81% increase) over 2 years and by $1.58 billion (80% increase) over 5 years (Fig 4). Most of the difference in costs over 5 years was due to increased spending on MDR-TB treatment (56% of the increase), followed by diagnostic tests (37% of the increase). Deploying *Truenat POC* instead of *Xpert* increased cumulative healthcare expenditures by $100 million (7% increase) over 2 years and by $270 million (8% increase) over 5 years. Most of the difference in costs over 5 years was due to increased spending on MDR-TB treatment (63% of the increase), followed by drug-susceptible TB treatment (22% of the increase).

## Discussion

A major WHO priority for TB diagnostics is to implement a rapid, sputum-based molecular test to replace smear microscopy at the peripheral level (i.e., microscopy centers and attached primary healthcare facilities) [7,8]. Our model-based analysis shows that in India, Truenat, when replacing smear microscopy and used at point-of-care, increases the number of TB cases correctly detected and linked to care by 590 per 10,000 individuals with presumptive TB. It also increases life expectancy by nearly 0.4 years and is cost-effective. While *Truenat DMC* was economically inefficient among the four strategies, it was cost-effective when compared directly to *SSM*. The cost-effectiveness of *Truenat POC*, compared to *SSM*, was consistent across a wide range of clinical and cost parameter values.

The WHO’s target product profile (TPP) of the “smear replacement test” includes a set of minimal and optimal requirements [7,8]. Truenat fits many minimal TPP standards, including battery-powered operation and <2 hours to result. However, it currently falls short of optimal TPP standards for test characteristics (i.e., sensitivity for TB detection ideally better than Xpert) and minimal standards for price (i.e., <$6 per reagent and <$1,400 per instrument) [7,8]. Our analysis shows that despite these limitations, *Truenat POC* increases life expectancy and is cost-effective compared to *SSM* or *Xpert*.

Our analysis, however, reveals an important relationship between TB detection sensitivity and linkage-to-care. As Truenat’s TB detection sensitivity decreases, the linkage-to-care level necessary for *Truenat POC* to be cost-effective compared to *Xpert* increases. This interplay is consistent with other modeling work showing that a theoretical, peripheral-level TB test with inferior performance characteristics but improved treatment initiation rates, compared to district-level Xpert testing, would reduce TB transmission and incidence [25]. Our results show that such a test can also be cost-effective. These findings together imply that increasing case detection (via improved test performance characteristics) and increasing the number of patients on life-prolonging treatment (via improved linkage-to-care) may yield synergistic results from a cost-effectiveness standpoint.

*Truenat POC* will be even more economically efficient if the price of the Truenat chip is reduced to 60% of its current estimate (with Truenat, therefore, becoming less expensive than Xpert). This reduced price would still be higher than the <$6 TPP stipulation. The 4-module Truelab system is also currently ten times more expensive than the TPP price stipulation for a test instrument. Even so, our scenario analysis shows *Truenat POC* is cost-effective, cost-saving, or decrementally cost-effective (“much less costly and almost as good”), relative to *Xpert*, across the wide range of parameter values considered.

Results from our scenario analysis are notable in that improvements in Truenat’s TB detection sensitivity and linkage-to-care yield favorable but higher ICERs. For example, *Truenat POC*, at 86% sensitivity and 90% linkage-to-care, is cost-saving (more life-years, lower cost) compared to *Xpert*, but becomes cost-effective (more life-years, higher cost) if linkage-to-care improves to 100%. This occurs because TB treatment costs, not diagnostic test costs, are the main driver of lifetime costs. Factors leading to higher treatment initiation increase the cumulative costs per patient. Our analysis, nonetheless, shows that this increase in cumulative costs is justified, from a cost-effectiveness perspective, by the clinical benefit of more patients receiving life-prolonging treatment (i.e., more life-years are saved at an acceptable additional cost). Such findings have also been observed in HIV screening programs, where a major driver of cost is the treatment pathway triggered when a previously undetected case is identified and the patient is linked to life-prolonging care [26,27].

The RNTCP can consider these findings as it pursues its ambitious National Strategic Plan (NSP) to eliminate TB in India by 2025 [3]. The NSP’s projected budget of $2.49 billion for the 2017–2020 period assumes approximately half of patients with TB receive molecular testing. Additional diagnostics are being planned for another 4.5 million patients during this period [3]. Our analysis shows that scaling up molecular diagnostics will increase the required budget but the majority of the cost will be from MDR-TB treatment. A recent economic analysis for India similarly found that full replacement of smear microscopy with Xpert would substantially increase budget requirements but would result in lower cost per MDR-TB case initiated on treatment [21]. As the RNTCP plans its NSP budget for 2020–2025, it should consider MDR-TB treatment costs as much as, if not more than, the prices of diagnostic tests.

A “real-world” economic evaluation by Vassall et al. showed that Xpert did not improve the cost-effectiveness of drug-susceptible TB diagnosis and treatment over a 6-month horizon, contrary to previous projections [28]. This finding underscores the need for cost-effectiveness analyses to account for uncertainties in implementation constraints [29]. In modeling our diagnostic and treatment pathway, we adjusted for many demand-side constraints, including care-seeking, test uptake, and adherence. We also adjusted for some supply-side constraints, including infrastructure limitations. While these parameters vary with geographical region (e.g., rural, urban, etc.), we drew on aggregated sources, including national surveillance data and studies using demographically and geographically representative population samples from India [2,4,6], to adjust for the “average” peripheral healthcare setting in India. We found that neither *Xpert* nor *Truenat POC* was cost-effective compared to *SSM* until 6 years after initial testing, well beyond the 6-month time horizon considered by Vassall et al [28].

Our findings apply to adult, HIV-negative individuals with presumptive pulmonary TB. In populations with high prevalence of undertreated HIV [28], the potential clinical benefits of *Xpert* and *Truenat POC* could be offset by high HIV-related mortality. HIV is also associated with lower sensitivity of TB diagnostics and substantial costs of screening and treatment [1,22,26,27]. Thus, studies of Truenat POC testing in this vulnerable population would be valuable.

We focused our analysis closely on the comparison of *Truenat POC* and *Xpert* because the decentralization of rapid, molecular TB diagnostics to the peripheral healthcare setting is currently of policy relevance to India and other high-burden countries [4,6,21,30,31]. We, however, did not consider a POC strategy for Xpert. Studies conducted primarily within well-resourced clinics in South Africa have demonstrated the feasibility of Xpert POC testing [32–36]. We are aware of only one study in India evaluating Xpert POC testing, within an outpatient clinic of a well-resourced tertiary hospital [31]. It remains unclear whether this strategy can be replicated universally across diverse settings, including rural and tribal/hilly areas of India [30,31,37]. While infrastructure concerns may have, to an extent, deterred interest in a POC strategy for Xpert in many high-burden countries [30,38], they have also spurred substantial research and development in a new generation of molecular diagnostics, such as Truenat, specifically intended for the primary healthcare setting [7,8]. Our study is focused on this latter area of policy interest.

Limitations of our study include those related to parameter inputs and model structure. We did not account for reduced transmission from faster diagnosis and treatment initiation, which could improve the cost-effectiveness of POC testing at a broader scale. Because preference weights for health states were not incorporated into the model, our study did not account for the additional benefits of reduced TB-related morbidity that could result from POC testing. We also did not consider some supply-side constraints that could disrupt successful POC testing and treatment, such as irregularities in test reagent and drug supply chains, potential lack of human resources, and variations in provider adherence to the diagnostic and treatment pathway [29]. Due to the lack of microcosting data regarding the unit costs of POC testing within the primary healthcare setting, our base case analysis was limited in its ability to adjust for the cost disadvantages of conducting tests at a lower operational scale, though we evaluated this in scenario analysis.

Importantly, more data are needed regarding Truenat test characteristics. In our analysis, the estimated sensitivity of Truenat for TB detection was based on a single study, and RIF-resistance detection performance was based on a clinical validation study conducted by the manufacturer. We, therefore, varied these parameters widely in sensitivity analyses. Test characteristics may improve with newer versions of Truenat under evaluation. Furthermore, as Truenat chips for other infectious diseases (e.g., HIV, malaria, HCV), which utilize the same Truelab system, become validated for India, the wide implementation of the Truenat platform could eventually help strengthen and improve the clinical and economic efficiency of the general health system.

Our study was limited to a health system perspective and does not include economic costs or savings to patients. Our healthcare expenditure projections should also be interpreted only for diagnostic test costs, drug costs, and treatment-associated clinic, monitoring, and hospitalization costs. We did not include “start-up” costs of establishing Xpert or Truenat in the field, such as costs of training staff to utilize the machines and costs of maintaining steady supply chains and reporting systems. Implementation studies are needed and ongoing to demonstrate Truenat’s efficacy under real-world conditions. As results become available, dedicated studies will be needed to estimate the implementation costs of Truenat on a larger scale, as there have been for Xpert [31,39–41].

Truenat is, nonetheless, the first TB test with capacity comparable to the 4-module Xpert but with operational features suited for the peripheral level. Our model-based analysis shows that, when used at point-of-care for TB diagnosis, Truenat improves linkage-to-care, increases life expectancy, and is cost-effective compared with smear microscopy or Xpert. Appropriate diagnostics are needed at every level of the healthcare system [8]. Truenat deployed at the peripheral level may be complementary to other diagnostic technologies, such as Xpert and Xpert Ultra [42], which are appropriate for the district and sub-district levels, and Xpert Omni and other compact, 1-module testing platforms, which may be valuable for community-based active case-finding (clinicaltrials.gov, NCT 03168945) [43]. In this way, Truenat should contribute to TB control and, thus, should be more widely utilized in India.

## Supporting information

S1 Appendix Technical appendix. (DOCX)
