# Supplementary material for: Rapid, point-of-care diagnosis of tuberculosis with novel Truenat assay: Cost-effectiveness analysis for India’s public sector
Source: PLoS One. 2019 Jul 2;14(7):e0218890. doi: 10.1371/journal.pone.0218890 (PMC6605662; doi:10.1371/journal.pone.0218890)
Supplement: S1 Appendix — (DOCX) [file pone.0218890.s001.docx]

**TECHNICAL APPENDIX**

**Rapid, point-of-care diagnosis of tuberculosis with novel Truenat assay:
Cost-effectiveness analysis for India’s public sector**

David J. Lee, Nagalingeswaran Kumarasamy, Stephen C. Resch, Gomathi N. Sivaramakrishnan, Kenneth H. Mayer, Srikanth Tripathy, A. David Paltiel, Kenneth A. Freedberg,
Krishna P. Reddy

**Contents**

S1. Methods: Additional Information p 2 S1.1 Analytic Overview p 2

S1.2 Model Overview p 2

S1.3 Base Case Input Parameters p 3

S1.4 Sensitivity and Scenario Analyses p 6

S1.5 Budget Impact Analysis p 7

Appendix References p 8

Table A p 13

Fig A p 14

Fig B p 15

Fig C p 16

Fig D p 17

Fig E p 18

Fig F p 19

**S1. Methods: Additional Information**

**S1.1 Analytic Overview**

***Diagnostic Strategies***

The sputum smear microscopy (*SSM*) strategy (Fig A) was modeled according to published national guidelines for India [1]. Patients provide two sputum samples, one collected at the time of testing initiation and the second collected the following morning. We assume that all patients are able to provide the first sputum sample, but only a proportion of patients return the following morning to provide the second sputum sample. If smear-negative, a proportion of patients undergo a multi-step clinical diagnostic algorithm, which includes an antibiotic trial and chest radiography. Patients with a positive smear or clinical diagnosis are started on first-line treatment if they have no history of tuberculosis (TB) treatment. Patients who receive a positive smear or clinical diagnosis and were previously treated for TB are considered at higher risk for drug-resistance. They receive additional culture and drug-susceptibility testing (C&DST) to test for multidrug-resistant TB (MDR-TB). If C&DST is positive for MDR-TB, they initiate second-line treatment. This traditional *SSM* algorithm was included in this study as a baseline strategy for consistent and transparent comparisons with other cost-effectiveness analyses of TB diagnostics [2–4].

For *Xpert* and *Truenat* strategies (Figs B-C), patients provide only one sputum sample at the time of testing initiation. As in the *SSM* strategy, we assume that all patients are able to provide this sample. Xpert tests simultaneously for active TB and for rifampicin (RIF)-resistance, whereas Truenat tests for RIF-resistance only after a positive TB result. With Xpert, there is an additional probability of test failure due to power- and temperature-related issues, as informed by results from a recent feasibility study of Xpert for designated microscopy centers (DMCs) in India [5]. Upon test failure, we assume patients return for repeat testing in the following month. Patients who receive a negative test result with Xpert or Truenat but retain high clinical suspicion for TB may receive confirmatory C&DST.

All strategies evaluated in our study share the same level of implementation (i.e., full-scale deployment) for the same population considered in our study (i.e., adult, HIV-negative individuals with presumptive pulmonary TB).

***Cost-effectiveness Threshold***

It is increasingly recognized that cost-effectiveness thresholds should account for the “opportunity cost” of forgone health benefits of not providing some interventions to fund others [6–11]. This is especially relevant for low-resource settings where there are substantial constraints against raising healthcare expenditures [6]. Woods et al. recommend opportunity-cost based thresholds of 1—51% the national annual gross domestic product (GDP) *per capita* for low/middle countries and 18—71% for middle/high income countries [6]. In considering these estimates for India, a lower middle-income country [12], we chose a cost-effectiveness threshold that is 50% of the GDP *per capita* of India in 2017. Therefore, a strategy was considered “cost-effective” if its ICER was less than US$990/year-of-life saved (YLS) [13].

**S1.2 Model Overview**

***TB Natural History***

Our model simulates the natural history, diagnosis, and treatment of TB (Fig D). We simulated cohorts of five million individuals to achieve stable per-person estimates. Individuals are in one of several possible TB “states” (uninfected, latent TB infection, active TB disease, previously treated TB, or uncompleted TB treatment) and may transition between these states in monthly cycles. Transition probabilities depend on disease- and treatment-related factors, including incidence of infection, symptom occurrence, relapse, and loss to follow-up (LTFU) during diagnostic testing or treatment. These probabilities are informed by literature and surveillance data. An individual also may transition to the “dead” state with a monthly probability that depends on the individual’s current TB state and treatment status.

For this analysis, we modeled a cohort of adult individuals with presumptive TB (i.e., >2 weeks of cough) who seek care and receive TB testing in India’s public sector. While all individuals are symptomatic at model entry, only some have true active TB disease. The prevalence of active TB disease in this cohort and the distribution of drug-susceptible tuberculosis (ds-TB) and MDR-TB are user-defined. All individuals, however, have a probability of developing active TB later in life, whether as a first infection and rapid progression (uninfected individuals), reactivation (latently infected individuals), or as reinfection and rapid progression or reactivation (previously infected individuals). Those without active TB also have a separate user-defined probability of developing symptoms suggestive of TB (but actually reflecting a disease other than TB) and may present to care for the initiation of TB testing.

As individuals transition monthly through “states” of TB progression and treatment, the model tracks clinical outcomes (e.g., cure, relapse, life-years accrued) and monthly TB-related healthcare costs (e.g., diagnostic tests, drugs, clinic visits). Throughout the simulation, all individuals are subject to age- and sex-stratified background mortality risks specific for India, while those with active, untreated TB have an excess mortality risk. Full model specifications can be found at http://www.massgeneral.org/mpec/cepac/.

***TB Diagnostic Testing***

When an individual with presumptive TB presents to care either at model entry or later in life, s/he is offered a user-defined sequence of TB diagnostic tests. For example, a patient who receives a negative test result (e.g., smear-negative) may be offered a follow-up confirmatory test (e.g., clinical diagnosis with chest radiography and antibiotic trial). For each test in the sequence, the patient has a probability of completing the test and a probability of retrieving the test result. The observed TB strain may differ from the patient’s true TB strain, due to suboptimal test characteristics.

***TB Treatment and Loss to Follow-up***

Patients who receive a positive test result for TB have a probability of linking to care. If successfully linked to care, patients begin a treatment regimen based on their observed TB strain’s resistance profile and history of TB treatment [14]. A first-line treatment regimen (rifampicin/isoniazid/pyrazinamide/ethambutol) is given to patients with observed ds-TB and no prior TB treatment. A retreatment regimen (rifampicin/isoniazid/pyrazinamide/ethambutol/streptomycin) is given to patients with observed ds-TB and prior TB treatment (“previously treated”). A second-line regimen (kanamycin/levofloxacin/ethionamide/cycloserine/pyrazinamide/ethambutol) is given to patients with observed MDR-TB, regardless of treatment history. Treatment regimens vary in duration (i.e., 6 months for first-line, 8 months for retreatment, and 24 months for second-line), during which there is a monthly probability of LTFU.

Because the observed TB strain may differ from the patient’s true TB strain, patients may be placed on an inappropriate treatment regimen (e.g., first-line regimen for MDR-TB). Such patients “fail” their treatment and are subject to the same mortality as those with untreated, active TB. Patients are monitored while receiving treatment, and, therefore, have a monthly probability of observing treatment failure and switching to the appropriate treatment regimen.

All patients have a monthly probability of LTFU during treatment. Because this is a monthly probability, patients who are on a longer treatment regimen (e.g., 24 months for MDR-TB treatment) have a greater overall risk of not completing their treatment course. Patients lost to follow-up, however, have a probability of being “cured” of active TB disease after receiving partial treatment, and this probability increases with the proportion of treatment duration completed before LTFU.

Taking these variables together, there are four possible treatment outcomes. First, those who successfully complete treatment (i.e., no LTFU) and achieve cure transition to the “previously treated” state. Second, those who complete treatment (i.e., no LTFU) but do not achieve cure remain in the “active” state. Third, those who are lost to follow-up but, nonetheless, achieve cure transition to the “uncompleted TB treatment” state. These individuals are at risk of developing resistance to the treatment regimen received. Fourth, those who are lost to follow-up before achieving cure remain in the “active” state. These individuals are at risk of developing resistance to the treatment regimen received. Those in either the “previously treated” or “uncompleted TB treatment” state may have recurrence of active TB disease due to either exogenous reinfection or endogenous relapse.

**S1.3 Base Case Input Parameters**

***TB Prevalence***

Data regarding the true prevalence of TB in India are limited. National TB prevalence surveys are being planned for 2019 [15]. Therefore, we used prevalence estimates by Khaparde et al. [16], based on the same cohort of individuals with presumptive TB in India as that in the Xpert implementation study [17]. The authors estimated the true prevalence of TB among individuals with presumptive TB using the test characteristics of sputum smear microscopy and Xpert and the proportion of bacteriologically confirmed cases in both phases of the implementation study. Their calculations can be found in the Supplement to their study [16]. Based on this method, the prevalence of TB was 15% among those not previously treated for TB and 27% among those previously treated for TB.

***Natural History***In our model, individuals have a monthly probability of becoming infected with a new TB strain and developing active pulmonary disease as a result. Data for this parameter are limited. While the World Health Organization (WHO) provides an estimate for the annual incidence of active TB cases (all forms) per year (i.e., 2.6 million for individuals age >15 years in 2016), it does not stratify this incidence by pulmonary versus extrapulmonary disease. It also does not stratify incidence by the source of infection—that is, infection from a new strain versus from relapse of an older strain that was in remission from previous treatment.

Therefore, to estimate our parameter of interest (i.e., monthly incidence of developing active pulmonary TB due to infection or reinfection with a new *Mycobacterium TB* strain), we subtracted from the WHO incidence the estimated burden of relapsed and extrapulmonary TB, as reported in literature. More specifically, among the 2.6 million new active TB cases for individuals ages >15 years, we assumed 16% were due to extrapulmonary disease [18], 91% of which occurred among adults [19]. Of the remaining pulmonary TB cases, we assumed that 14% were recurrent cases [20], 69% of which are due to relapse [21,22]. Given the lack of age-stratified data, we assumed that the relapse rate was equal for pediatric and adult cases. After subtracting these rates, we estimated that the monthly probability of developing active pulmonary TB due to infection, or reinfection for previously treated individuals, with a new *Mycobacterium tuberculosis* strain was 0.02% (Table A). [17–20,22–29]

Other parameters related to the natural history of TB, including the monthly mortality risk from untreated TB, are provided in Table A.

***Presumptive TB***

In our model, individuals undergo TB testing if they (1) develop symptoms suggestive of TB (i.e., >2 or more weeks of cough) and (2) subsequently seek medical care for their symptoms. We assumed all individuals with active TB remained symptomatic while in the active TB state. The monthly probability of seeking medical care among these individuals was 8%. This probability was derived from results of a recent national-level analysis [25].

Individuals who do not have active TB (i.e., uninfected or previously treated individuals) also have a monthly probability of developing symptoms suggestive of TB and seeking medical care. Their symptoms are due to causes other than TB, such as bacterial pneumonia. Data regarding this parameter are lacking. However, an estimate can be derived by, first, estimating the incidence of individuals (with and without active TB) in the population who develop symptoms suggestive of TB and seek medical care (variable 1) and, second, subtracting from this estimate the proportion of individuals who truly have active TB (variable 2). The Xpert implementation study provides an estimate for variable 1, based on the total number of individuals who were tested for TB at selected DMCs and the total person-years in the DMCs’ catchment areas over the study period [17]. Using data from the Xpert implementation study, we then estimated variable 2 by applying the method used by Khaparde et al., which was described in the prior section regarding TB prevalence [16]. Subtracting variable 2 from variable 1, the monthly probability of developing symptoms suggestive of TB and seeking medical care, among individuals without active TB, was 0.04%.

***Diagnostic Tests***

In the *SSM* strategy, the proportion of patients who undergo clinical diagnostic testing (e.g., antibiotic trial and chest radiography) after a smear-negative result was 39% [25]. In *Xpert* and *Truenat* strategies, patients who receive a negative test result with Xpert or Truenat may also undergo additional testing (i.e., confirmatory C&DST). However, given that Xpert and Truenat have higher sensitivity than sputum smear [30–32], we assumed that the proportion of individuals who receive additional testing after a negative Xpert or Truenat result was half of that among those with a negative smear result (Table A).

Also, in the *SSM* strategy, patients who are smear-positive and were previously treated for TB may provide an additional sputum sample to test for MDR-TB with C&DST (Fig A). We assumed that the proportion of smear-positive patients who submit this additional sputum sample was 75%. This was based on the Xpert implementation study in India, in which 75% of patients submitted an additional sputum sample for C&DST when requested [17].

We are aware of two validation studies of Truenat’s performance characteristics for TB detection, both of which were conducted in India [31,32]. However, there are currently no published studies on Truenat’s performance characteristics for RIF‑resistance detection. The manufacturer reports a clinical validation study, based at a TB referral center in India, in which 115 TB positive sputum samples were tested by both a Line Probe Assay (LPA) and Truenat for RIF-resistance detection [33]. Using LPA as the gold standard, Truenat’s sensitivity and specificity for RIF-resistance detection was 94% and 98%. These values were used for the base case, and parameters were varied widely in sensitivity analyses.

***Linkage-to-Care***

Patients diagnosed with pulmonary TB in DMCs are referred to healthcare facilities close to their homes for treatment [1,25]. Patients who complete this referral process and initiate treatment are successfully “linked to care.” A recent national-level analysis of the TB “cascade of care” estimated that the overall linkage‑to‑care rate among smear-positive patients diagnosed at DMCs is 84% (95% confidence interval, 80–88%) [25]. This analysis also showed that the majority of this 16% gap between diagnosis and treatment initiation—sometimes referred to as “pretreatment loss to follow-up”—occurs during the process of referral from DMCs to healthcare facilities closer to patients’ homes [5,17,25,34–37]. Therefore, while some primary healthcare facilities also function as DMCs, our study focuses on referral cases. We also assumed that this 16% gap between diagnosis and treatment initiation is the same for any sputum-based test conducted in DMCs, whether smear microscopy, Xpert, or Truenat.

Given the novelty of the Truenat assay, there are currently no published data on the linkage-to-care of individuals diagnosed via point-of-care (POC) testing with Truenat in India. However, as a proxy, we drew upon data from trials and implementation studies of POC testing with Xpert in the primary healthcare setting in high TB burden countries [38–41]. Overall, these data suggest that POC testing with Xpert improves linkage-to-care. For example, the TB-NEAT study [38], a randomized, controlled trial (RCT) of Xpert within peri-urban primary care clinics, showed that 97% of patients who received a positive TB result with POC Xpert testing started treatment within one week. In contrast, the XTEND study [42], an RCT of Xpert in a laboratory setting, showed that only 83% of all patients who received a positive TB result from lab-based Xpert testing initiated treatment.

Two studies directly compared lab-based Xpert testing to POC Xpert testing in a primary healthcare setting. One prospective study showed that 95% of individuals who received a positive TB result on POC Xpert testing initiated treatment (median time to treatment, 0 days), compared to 87% of individuals who received a positive TB result via laboratory-based Xpert testing (median time to treatment, 5 days) [39]. Similarly, a recent RCT showed that 96% of patients who received a positive TB result via POC Xpert testing in rural primary care clinics in South Africa initiated treatment within 30 days, compared to 90% of those who received a positive TB result via lab-based Xpert testing in the same time period [40].

We are aware of only one study that evaluated POC Xpert testing in India [41]. This study took place in an outpatient clinic of a tertiary care hospital. Among patients who received a positive TB result and whose follow-up data were available, all initiated TB treatment. However, same-day treatment initiation was limited to only those individuals who lived near the tertiary care hospital and did not need referral to a more local treatment center.

Taking these data together, we assumed linkage-to-care for individuals diagnosed with Truenat in the primary healthcare setting (*Truenat POC*) was 95% within one month of diagnosis. Other modeling work evaluating a theoretical “peripheral nucleic acid amplification test (NAAT) test” to replace sputum smear microscopy for the South East Asia Region have also used this value [43]. We did not assume that POC testing with Truenat would lead to same-day linkage-to-care, as this value would be substantially lower than 95%.

***Costs***

We derived the unit costs of diagnostic tests from a microcosting study by Rupert et al. [44]. In this study, the mean unit costs of sputum smear microscopy and Xpert were calculated using data from DMCs that were purposely chosen to account for regional variations in India. For sputum smear microscopy, the average unit costs were $0.18 (overhead), $0.04 (building space), $0.004 (equipment), $0.06 (labor), $0.30 (reagents and chemicals), and $0.25 (consumables) (see Table 3 in the Rupert et al. study). For Xpert, the average unit costs were $0.26 (overhead), $0.03 (building space), $1.26 (equipment), $0.05 (labor), $10.44 (reagents and chemicals, i.e., cartridge), and $0.25 (consumables) (see Table 4 in the Rupert et al. study). For Truenat, the unit costs of the equipment (test instruments) and reagents and chemicals (Truenat chip) were obtained from the manufacturer (see Methods of the main text). We assumed that all other unit costs of Truenat, including overhead and labor, were similar to those of Xpert.

The unit costs were summed to determine the total cost per test. Because the Rupert et al. study reports costs in 2014 US dollars (USD), we adjusted costs to 2017 USD by applying methods from Shah et al. [45]. Specifically, the proportion of total costs that are non‑tradeable (domestic) were adjusted using Indian inflation rates, and the proportion of total costs that are tradeable (imported and exported) were adjusted using US inflation rates.

For TB treatment, we used published national guidelines and epidemiological data to determine the cost components for each treatment regimen [1,14,46,47]. These components included drugs, clinic visits, monitoring tests, and expected hospitalizations during treatment. For example, first-line therapy for ds-TB includes 24 outpatient visits during the intensive phase of treatment, in which patients receive isoniazid/rifampicin/pyrazinamide/ethambutol, and 16 outpatient clinic visits during the continuation phase, in which patients receive isoniazid/rifampicin/ethambutol. We assumed a daily dose for an individual weighing 55—69 kg. To monitor response to first-line therapy, two sputum samples are collected for smear microscopy at the end of the intensive phase, two months into the continuation phase, and upon treatment completion. We also assumed 7.5% of TB patients would be hospitalized during first-line therapy, for a mean duration of 30 days [46].

These components and their expected quantities were multiplied by their respective unit costs. Unit costs for drugs were based on prices offered by the Global Drug Facility (GDF) [48], while unit costs for outpatient visits and hospitalizations were based on WHO-CHOICE [49]. Unit costs for monitoring tests (i.e., sputum smear microscopy, culture, and drug-susceptibility testing) are provided in Table 1 of the main text [44]. Dividing the final sum of costs of each treatment regimen by the regimen’s expected duration (i.e., 6 months for first-line, 8 months for retreatment, and 24 months for second-line), the monthly costs of TB treatment were $28.13 (first-line), $32.25 (retreatment), and $104.23 (second-line). As with diagnostic test costs, treatment costs were adjusted to 2017 USD using Indian inflation rates for the proportion of costs that are non‑tradeable (e.g., outpatient visits, hospitalizations) and US inflation rates for the proportion of total costs that are tradeable (e.g., drugs).

Our study did not include costs associated with tax and distribution of drugs and diagnostics. Because the Truenat platform is manufactured locally in India, however, it would not be subjected to importation-related tax and distribution costs for India. We also did not incorporate “start-up” costs of establishing and maintaining Xpert or Truenat in the field. For example, we did not include the cost of training new staff to utilize the test instruments. The number of test instruments needed to be deployed may also differ for Xpert and Truenat as they may be intended for different settings (e.g., DMCs or primary healthcare facilities). These settings may require different operational systems to maintain Truenat and Xpert in the field, such as a steady supply chain for cartridges (Xpert) and chips (Truenat), and to report test results to national surveillance programs. Together, these new machines and operational systems may require additional supervision and quality control. Dedicated studies will be needed to estimate such start-up costs for Truenat, as there have been for Xpert [50–53].

**S1.4 Sensitivity and Scenario Analyses**

***Empirical Treatment***

Under the *SSM* strategy, smear-negative individuals may be started on empirical TB treatment after undergoing a clinical diagnostic process, which includes an antibiotic trial and chest radiography. Molecular diagnostics like Xpert and Truenat may reduce the need for empirical treatment due to their higher sensitivity, which may increase clinicians’ confidence in a negative test result [54]. However, the extent to which the utilization of empirical treatment will change with Xpert and Truenat is currently unknown. One study based in a South African hospital showed that after the implementation of Xpert, the proportion of patients who were started on empirical TB treatment decreased from 79% to 28% [55]. The TB-NEAT study, a randomized controlled trial of Xpert in the primary care setting, however, showed that the reduction in the proportion of patients started on empirical treatment was not as prominent (i.e., 26% in the sputum smear microscopy groups versus 17% in the Xpert group) [38].

These studies were conducted in HIV-endemic settings, where providing empirical TB treatment is generally more common due to higher TB-related mortality [54]. Though our analysis focused on an HIV-negative population, we also evaluated a scenario in which empirical treatment is offered to a proportion of individuals who receive a negative Xpert or Truenat result and to at least as high a proportion for individuals who receive a negative sputum smear result. More specifically, for this scenario analysis, we assumed all individuals who receive a negative smear result undergo clinical diagnostic testing (i.e., antibiotic trial and chest radiography). Based on the test characteristics of clinical diagnostic testing, we estimated that 16% of these individuals would be offered empirical treatment. We assumed that the same proportion of individuals receiving a negative Xpert or Truenat result would be offered empirical treatment. Linkage-to-care for empirical treatment remained 84% for DMC-based tests and 95% for POC testing (i.e., *Truenat POC*).

***Test Volume***

DMCs across geographically and demographically diverse regions in India are often each linked to as many as 5 primary healthcare facilities [5,17]. This theoretically may suggest that the test volume capacity of a DMC is at least 5‑fold higher than that of a single primary healthcare facility such that the DMC is able to simultaneously handle test referrals from all 5 of its linked facilities. Nonetheless, robust data regarding the test volume capacity of DMCs compared to primary healthcare facilities are lacking for India. In South Africa, however, a multi-center randomized, controlled trial of POC testing with Xpert in primary healthcare facilities has allowed for this comparison [38,56]. Specifically, Pooran et al. showed that a centralized laboratory performing TB tests with Xpert had test volumes 2—5 times higher than those of primary healthcare facilities performing POC testing with Xpert [56]. Using the upper end of this scale as a proxy for our analysis, we considered the scenario in which DMCs on average have test volumes that are 5‑fold higher than those of primary healthcare facilities in India. We also considered a 10-fold scale in view of the very high test volumes that some DMCs experience in certain settings like a mega-city [37,57].

The overall cost of operating the Truenat platform includes various cost categories, some of which may vary and others which may not vary with the test volume of a facility. While “variable costs” like reagents and chemicals (Truenat chip) and consumables vary linearly with the test volume of the facility, “fixed costs” like overhead, building space, and equipment (test instruments) remain independent of the test volume. Certain elements of labor, such as the daily set-up and shut-down of the testing platform, maintenance and calibration of the test system, and running of tests in batches, also have fixed costs, which we assumed to represent 25% of the overall labor cost. Taken together, fixed component costs (overhead, building space, equipment, and 25% of labor) represented 4% of the per‑test cost of operating the Truenat platform in DMCs. Derivations of component costs are provided in the Methods sections of the main text and this appendix.

In primary healthcare facilities, where there are lower test volumes, fixed costs are spread over fewer tests, resulting in a higher per‑test cost of operating the Truenat platform. We estimated that when primary healthcare facilities have test volumes 5‑ and 10‑fold lower than those of DMCs, the per‑test cost of *Truenat POC* increased from $13.20 (base case) to $15.32 and $17.96. We evaluated the cost-effectiveness of *Truenat POC* at these higher costs.

In this scenario analysis, fixed component costs increased proportionally to the change in test volumes of DMCs versus primary healthcare facilities. We also assumed that the per square meter cost of building space and labor rates were the same for both settings. Because these costs may, in fact, be lower for primary healthcare facilities than for DMCs, our calculations conservatively overestimate the per‑test costs of *Truenat POC*.

***Truenat Chip Cost***

Because the public sector cost of Truenat may decrease based on volume commitment by the Indian government, we conducted a scenario analysis in which the cost of the Truenat chip was negotiated to 60% of the current estimate. This number was chosen based on the historic precedent of price negotiations for the Xpert cartridge, in which a volume commitment of >3 million cartridges per year reduced Xpert’s cartridge price to 60% of its base price for India and other approved countries [4,58].

**S1.5 Budget Impact Analysis**

We assumed 7.9 million patients with presumptive TB would be tested every year, based on the incidence estimate reported in the Xpert implementation study in India and the population size of individuals aged >15 years in 2016 [17,59]. Because the implementation study did not stratify incidence by age group, we assumed that TB incidence in adults is the same as that in the general population.

**Appendix References**

1. Central TB Division. Revised National Tuberculosis Control Programme: training module for medical practitioners [Internet]. New Delhi, India: Directorate General of Health Services, Ministry of Health and Family Welfare; 2010. Available: https://tbcindia.gov.in/showfile.php?lid=2908

2. Menzies NA, Gomez GB, Bozzani F, Chatterjee S, Foster N, Baena IG, et al. Cost-effectiveness and resource implications of aggressive action on tuberculosis in China, India, and South Africa: a combined analysis of nine models. Lancet Glob Health. 2016;4: e816–e826. doi:10.1016/S2214-109X(16)30265-0

3. Vassall A, Siapka M, Foster N, Cunnama L, Ramma L, Fielding K, et al. Cost-effectiveness of Xpert MTB/RIF for tuberculosis diagnosis in South Africa: a real-world cost analysis and economic evaluation. Lancet Glob Health. 2017;5: e710–e719. doi:10.1016/S2214-109X(17)30205-X

4. Vassall A, Kampen S van, Sohn H, Michael JS, John KR, Boon S den, et al. Rapid diagnosis of tuberculosis with the Xpert MTB/RIF assay in high-burden countries: a cost-effectiveness analysis. PLoS Med. 2011;8: e1001120. doi:10.1371/journal.pmed.1001120

5. Raizada N, Sachdeva KS, Sreenivas A, Vadera B, Gupta RS, Parmar M, et al. Feasibility of decentralised deployment of Xpert MTB/RIF test at lower level of health system in India. PLoS One. 2014;9: e89301. doi:10.1371/journal.pone.0089301

6. Woods B, Revill P, Sculpher M, Claxton K. Country-level cost-effectiveness thresholds: initial estimates and the need for further research. Value Health. 2016;19: 929–935. doi:10.1016/j.jval.2016.02.017

7. Ochalek JM, Lomas J, Claxton KP. Cost per DALY averted thresholds for low- and middle-income countries: evidence from cross country data. [Internet]. York, UK: Centre for Health Economics, University of York; 2015 pp. 1–50. Report No.: 122. Available: https://pure.york.ac.uk/portal/en/publications/cost-per-daly-averted-thresholds-for-low-and-middleincome-countries(12487fa5-e63f-4ac3-9fa4-03b2795065eb).html

8. Drummond MF, Schulpher MJ, Claxton K, Stoddart GL, Torrance GW. Methods for the economic evaluation of health care programmes. 4th ed. Oxford, UK: Oxford University press; 2015.

9. Claxton K, Walker S, Palmer S, Sculpher M. Appropriate perspectives for health care decisions [Internet]. York, UK: Centre for Health Economics, University of York; 2013. Report No.: 54. Available: http://www.york.ac.uk/media/che/documents/papers/researchpapers/rp54_appropriate_perspectives_for_health_care_decisions.pdf

10. Claxton K, Martin S, Soares M, Rice N, Spackman E, Hinde S, et al. Methods for the estimation of the National Institute for Health and Care Excellence cost-effectiveness threshold. Health Technol Assess. 2015;19: 1–503, v–vi. doi:10.3310/hta19140

11. Bertram MY, Lauer JA, De Joncheere K, Edejer T, Hutubessy R, Kieny M-P, et al. Cost-effectiveness thresholds: pros and cons. Bull World Health Organ. 2016;94: 925–930. doi:10.2471/BLT.15.164418

12. World Bank. Data: World Bank country and lending groups [Internet]. Washington, DC, USA: The World Bank; 2018. Available: https://datahelpdesk.worldbank.org/knowledgebase/articles/906519-world-bank-country-and-lending-groups

13. International Monetary Fund. World economic and financial surveys: world economic outlook database [Internet]. International Monetary Fund; 2018. Available: http://www.imf.org/external/pubs/ft/weo/2018/01/weodata/index.aspx

14. Central TB Division. RNTCP: technical and operational guidelines for tuberculosis control in India, 2016 [Internet]. New Delhi: Directorate General of Health Services, Ministry of Health and Family Welfare; 2016. Available: https://tbcindia.gov.in/index.php

15. World Health Organization. Global tuberculosis report 2018 [Internet]. Geneva, Switzerland: World Health Organization; 2018. Available: http://www.who.int/tb/publications/global_report/en/

16. Khaparde S, Raizada N, Nair SA, Denkinger C, Sachdeva KS, Paramasivan CN, et al. Scaling-up the Xpert MTB/RIF assay for the detection of tuberculosis and rifampicin resistance in India: an economic analysis. PLoS One. 2017;12: e0184270. doi:10.1371/journal.pone.0184270

17. Sachdeva KS, Raizada N, Sreenivas A, Hoog AH van’t, Hof S van den, Dewan PK, et al. Use of Xpert MTB/RIF in decentralized public health settings and its effect on pulmonary TB and DR-TB case finding in India. PLoS One. 2015;10: e0126065. doi:10.1371/journal.pone.0126065

18. World Health Organization. Global tuberculosis report 2017 [Internet]. Geneva, Switzerland: World Health Organization; 2017. Available: http://www.who.int/tb/publications/global_report/en/

19. Prakasha SR, Suresh G, D’sa IP, Shetty SS, Kumar SG. Mapping the pattern and trends of extrapulmonary tuberculosis. J Glob Infect Dis. 2013;5: 54. doi:10.4103/0974-777X.112277

20. World Health Organization, Regional Office for South-East Asia. Tuberculosis control in the South-East Asia Region: annual report 2016 [Internet]. New Delhi, India: World Health Organization; 2016. Available: http://apps.who.int/iris/handle/10665/205286

21. Sahadevan R, Narayanan S, Paramasivan CN, Prabhakar R, Narayanan PR. Restriction fragment length polymorphism typing of clinical isolates of Mycobacterium tuberculosis from patients with pulmonary tuberculosis in Madras, India, by use of direct-repeat probe. J Clin Microbiol. 1995;33: 3037–3039.

22. De S. High relapse rate in RNTCP: An increasing concern and time to intervene [letter to editor]. Lung India. 2013;30: 85. doi:10.4103/0970-2113.106129

23. Narayanan S, Swaminathan S, Supply P, Shanmugam S, Narendran G, Hari L, et al. Impact of HIV infection on the recurrence of tuberculosis in South India. J Infect Dis. 2010;201: 691–703. doi:10.1086/650528

24. Sharma SK, Ryan H, Khaparde S, Sachdeva KS, Singh AD, Mohan A, et al. Index-TB guidelines: guidelines on extrapulmonary tuberculosis for India. Indian J Med Res. 2017;145: 448. doi:10.4103/ijmr.IJMR_1950_16

25. Subbaraman R, Nathavitharana RR, Satyanarayana S, Pai M, Thomas BE, Chadha VK, et al. The tuberculosis cascade of care in India’s public sector: a systematic review and meta-analysis. PLoS Med. 2016;13: e1002149. doi:10.1371/journal.pmed.1002149

26. Tiemersma EW, van der Werf MJ, Borgdorff MW, Williams BG, Nagelkerke NJD. Natural history of tuberculosis: duration and fatality of untreated pulmonary tuberculosis in HIV negative patients: a systematic review. PLoS One. 2011;6. doi:10.1371/journal.pone.0017601

27. Millington KA, Gooding S, Hinks TSC, Reynolds DJM, Lalvani A. Mycobacterium tuberculosis-specific cellular immune profiles suggest bacillary persistence decades after spontaneous cure in untreated tuberculosis. J Infect Dis. 2010;202: 1685–1689. doi:10.1086/656772

28. Central TB Division. TB India 2017: Revised National Tuberculosis Control Programme: annual status report [Internet]. New Delhi, India: Directorate General of Health Services, Ministry of Health and Family Welfare; 2017. Available: https://tbcindia.gov.in/index1.php?lang=1&level=2&sublinkid=4728&lid=3275

29. Sharma SK, Kumar S, Saha PK, George N, Arora SK, Gupta D, et al. Prevalence of multidrug-resistant tuberculosis among category II pulmonary tuberculosis patients. Indian J Med Res. 2011;133: 312–315.

30. Steingart KR, Schiller I, Horne DJ, Pai M, Boehme CC, Dendukuri N. Xpert® MTB/RIF assay for pulmonary tuberculosis and rifampicin resistance in adults. Cochrane Database Syst Rev. 2014;1: CD009593. doi:10.1002/14651858.CD009593.pub3

31. Nikam C, Kazi M, Nair C, Jaggannath M, M M, R V, et al. Evaluation of the Indian TrueNAT micro RT-PCR device with GeneXpert for case detection of pulmonary tuberculosis. Int J Mycobacteriol. 2014;3: 205–210. doi:10.1016/j.ijmyco.2014.04.003

32. Nikam C, Jagannath M, Narayanan MM, Ramanabhiraman V, Kazi M, Shetty A, et al. Rapid diagnosis of Mycobacterium tuberculosis with Truenat MTB: a near-care approach. PLoS One. 2013;8: e51121. doi:10.1371/journal.pone.0051121

33. Molbio Diagnostics Pvt. Ltd. Truenat MTB-RIf Dx: chip-based real time PCR test for Rifampicin resistant Mycobacterium tuberculosis [Internet]. Goa, India: Molbio Diagnostics Pvt. Ltd.; Available: http://molbiodiagnostics.com/packinserts/new/Truenat_MTB_RIF_Dx.pdf

34. Mehra D, Kaushik RM, Kaushik R, Rawat J, Kakkar R. Initial default among sputum-positive pulmonary TB patients at a referral hospital in Uttarakhand, India. Trans R Soc Trop Med Hyg. 2013;107: 558–565. doi:10.1093/trstmh/trt065

35. Khandekar J, Acharya AS, R TH, Sharma A. Do patients with tuberculosis referred from a tertiary care referral centre reach their peripheral health institution? Natl Med J India. 2013;26: 332–334.

36. Pillai D, Purty AJ, Prabakaran S, Singh Z, Soundappan G, Anandan V. Initial default among tuberculosis patients diagnosed in selected medical colleges of Puducherry: issues and possible interventions. Int J Med Sci Public Health. 2015;4: 957–960.

37. Thomas BE, Subbaraman R, Sellappan S, Suresh C, Lavanya J, Lincy S, et al. Pretreatment loss to follow-up of tuberculosis patients in Chennai, India: a cohort study with implications for health systems strengthening. BMC Infect Dis. 2018;18: 142. doi:10.1186/s12879-018-3039-3

38. Theron G, Zijenah L, Chanda D, Clowes P, Rachow A, Lesosky M, et al. Feasibility, accuracy, and clinical effect of point-of-care Xpert MTB/RIF testing for tuberculosis in primary-care settings in Africa: a multicentre, randomised, controlled trial. Lancet. 2014;383: 424–435. doi:10.1016/S0140-6736(13)62073-5

39. Hanrahan CF, Clouse K, Bassett J, Mutunga L, Selibas K, Stevens W, et al. The patient impact of point-of-care vs. laboratory placement of Xpert® MTB/RIF. Int J Tuberc Lung Dis. 2015;19: 811–816. doi:10.5588/ijtld.15.0013

40. Lessells RJ, Cooke GS, McGrath N, Nicol MP, Newell M-L, Godfrey-Faussett P. Impact of point-of-care Xpert MTB/RIF on tuberculosis treatment initiation: a cluster-randomized trial. Am J Respir Crit Care Med. 2017;196: 901–910. doi:10.1164/rccm.201702-0278OC

41. Schumacher SG, Thangakunam B, Denkinger CM, Oliver AA, Shakti KB, Qin ZZ, et al. Impact of point-of-care implementation of Xpert® MTB/RIF: product vs. process innovation. Int J Tuberc Lung Dis. 2015;19: 1084–1090. doi:10.5588/ijtld.15.0120

42. Churchyard GJ, Stevens WS, Mametja LD, McCarthy KM, Chihota V, Nicol MP, et al. Xpert MTB/RIF versus sputum microscopy as the initial diagnostic test for tuberculosis: a cluster-randomised trial embedded in South African roll-out of Xpert MTB/RIF. Lancet Glob Health. 2015;3: e450–e457. doi:10.1016/S2214-109X(15)00100-X

43. Sun AY, Pai M, Salje H, Satyanarayana S, Deo S, Dowdy DW. Modeling the impact of alternative strategies for rapid molecular diagnosis of tuberculosis in Southeast Asia. Am J Epidemiol. 2013;178: 1740–1749. doi:10.1093/aje/kwt210

44. Rupert S, Vassall A, Raizada N, Khaparde SD, Boehme C, Salhotra VS, et al. Bottom-up or top-down: unit cost estimation of tuberculosis diagnostic tests in India. Int J Tuberc Lung Dis. 2017;21: 375–380. doi:10.5588/ijtld.16.0496

45. Shah M, Chihota V, Coetzee G, Churchyard G, Dorman SE. Comparison of laboratory costs of rapid molecular tests and conventional diagnostics for detection of tuberculosis and drug-resistant tuberculosis in South Africa. BMC Infect Dis. 2013;13: 352. doi:10.1186/1471-2334-13-352

46. Goodchild M, Sahu S, Wares F, Dewan P, Shukla RS, Chauhan LS, et al. A cost-benefit analysis of scaling up tuberculosis control in India. Int J Tuberc Lung Dis. 2011;15: 358–362.

47. Central TB Division. Revised National Tuberculosis Control Programme DOTS-Plus guidelines [Internet]. New Delhi, India: Directorate General of Health Services, Ministry of Health and Family Welfare; 2010. Available: http://health.bih.nic.in/Docs/Guidelines/Guidelines-DOTS-Plus.pdf

48. Global Drug Facility. Global Drug Facility: product catalogue, 2016 [Internet]. Vernier, Switzerland: Global Drug Facility, Stop TB Partnership; 2016. Available: http://www.stoptb.org/assets/documents/gdf/drugsupply/GDF%20product%20catalog_25%20Jul%202016_final.pdf

49. World Health Organization. CHOosing Interventions that are Cost Effective (WHO-CHOICE): country-specific unit costs [Internet]. Geneva, Switzerland: World Health Organization; 2008. Available: http://www.who.int/choice/country/country_specific/en/

50. Hsiang E, Little KM, Haguma P, Hanrahan CF, Katamba A, Cattamanchi A, et al. Higher cost of implementing Xpert® MTB/RIF in Ugandan peripheral settings: implications for cost-effectiveness. Int J Tuberc Lung Dis. 2016;20: 1212–1218. doi:10.5588/ijtld.16.0200

51. Schnippel K, Meyer‐Rath G, Long L, MacLeod W, Sanne I, Stevens WS, et al. Scaling up Xpert MTB/RIF technology: the costs of laboratory- vs. clinic-based roll-out in South Africa. Trop Med Int Health. 2012;17: 1142–1151. doi:10.1111/j.1365-3156.2012.03028.x

52. Albert H, Nathavitharana RR, Isaacs C, Pai M, Denkinger CM, Boehme CC. Development, roll-out and impact of Xpert MTB/RIF for tuberculosis: what lessons have we learnt and how can we do better? Eur Respir J. 2016;48: 516–525. doi:10.1183/13993003.00543-2016

53. Abdurrahman ST, Emenyonu N, Obasanya OJ, Lawson L, Dacombe R, Muhammad M, et al. The hidden costs of installing Xpert machines in a tuberculosis high-burden country: experiences from Nigeria. Pan Afr Med J. 2014;18: 1–5. doi:10.11604/pamj.2014.18.277.3906

54. Theron G, Peter J, Dowdy D, Langley I, Squire SB, Dheda K. Do high rates of empirical treatment undermine the potential effect of new diagnostic tests for tuberculosis in high-burden settings? Lancet Infect Dis. 2014;14: 527–532. doi:10.1016/S1473-3099(13)70360-8

55. Theron G, Peter J, Meldau R, Khalfey H, Gina P, Matinyena B, et al. Accuracy and impact of Xpert MTB/RIF for the diagnosis of smear-negative or sputum-scarce tuberculosis using bronchoalveolar lavage fluid. Thorax. 2013;68: 1043–1051. doi:10.1136/thoraxjnl-2013-203485

56. Pooran A, Theron G, Zijenah L, Chanda D, Clowes P, Mwenge L, et al. Point of care Xpert MTB/RIF versus smear microscopy for tuberculosis diagnosis in southern African primary care clinics: a multicentre economic evaluation. Lancet Glob Health. 2019;7: e798–e807. doi:10.1016/S2214-109X(19)30164-0

57. Subbaraman R, Thomas BE, Sellappan S, Suresh C, Jayabal L, Lincy S, et al. Tuberculosis patients in an Indian mega-city: where do they live and where are they diagnosed? PLoS One. 2017;12: e0183240. doi:10.1371/journal.pone.0183240

58. Mirzayev F. Current dynamics in the Xpert MTB/RIF assay pricing mechanisms. Annecy, France: Xpert MTB/RIF Early Implementers Meeting; 2012. Available: http://www.stoptb.org/wg/gli/assets/html/day%202/Mirzayev%20-%20Xpert%20cartridge%20price%20dynamics.pdf

59. United Nations. World population prospects: the 2017 revision [Internet]. New York, USA: Population Division, Department of Economic and Social Affairs, United Nations; 2017. Available: https://esa.un.org/unpd/wpp/

**Table A. Additional model input parameters for model-based cost-effectiveness analysis of TB diagnostic strategies for patients with presumptive TB in India.**

| **Parameter** | **Base case** | **Range**^a^ | **References** |
| --- | --- | --- | --- |
| **Natural history** |  |  |  |
| Monthly probability of new active TB | 0.02% | 0.008 – 0.03% | [18–20,22–24] |
| Monthly probability of seeking medical care among people with active TB | 8% | 6 – 10% | [25] |
| Monthly probability of seeking medical care for symptoms suggestive of  TB among people who do not have active TB | 0.04% | 0.02 – 0.07% | Assumption |
| Monthly probability of death from untreated TB | 1.3% | 0.9 – 2.3% | [26] |
| Duration of active TB until self-cure, years | 2 | 1 – 3 | [27] |
| **Diagnostic tests (additional)** |  |  |  |
| Proportion of smear-positive patients previously treated for TB  who provide additional sputum for C&DST | 75% | 38 – 100% | Assumption |
| Proportion of Xpert- and Truenat-negative patients who undergo confirmatory  C&DST due to high clinical suspicion for TB^b^ | 20% | 0 – 40% | Assumption |
| **Treatment for TB (additional)** |  |  |  |
| Monthly probability of observing treatment failure during first-line therapy and  switching to second-line therapy | 3% | 0 – 21%^c^ | [28] |
| Monthly probability of observing treatment failure during retreatment therapy and  switching to second-line therapy | 5% | 2 – 21%^c^ | [28] |
| Probability of developing resistance to first-line and retreatment regimen after  loss to follow-up^d^ | 24% | 14 – 49% | [29] |
| Probability of developing resistance to first-line and retreatment regimen after  treatment failure | 19% | 14 – 49% | [29] |

Abbreviations: TB: tuberculosis. C&DST: culture and drug-susceptibility

^a^Range used for univariate sensitivity analysis.

^b^In both *Xpert* and *Truenat* strategies (*DMC* and *POC*), patients who receive a negative test result but retain high clinical suspicion for TB may receive confirmatory C&DST.

^c^Range based on variation across states [28].

^c^Probability that patient’s drug-susceptible TB strain will become resistant to first-line regimen and progress to multidrug-resistant TB strain after a patient is lost to follow-up during treatment. The probability that multidrug-resistant TB will become resistant to the second-line regimen is assumed to be the same value.

**Fig A. Diagnostic and treatment algorithm for sputum smear microscopy strategy (*SSM*).**


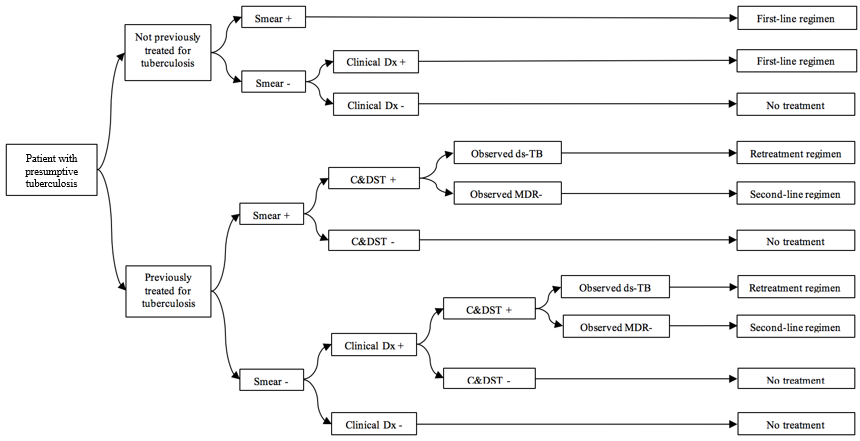


Abbreviations: Clinical Dx: clinical diagnosis. C&DST: culture and drug-susceptibility testing. TB: tuberculosis. ds-TB: drug-susceptible tuberculosis.
MDR-TB: multidrug-resistant tuberculosis.

Schematic shows the possible combination of tests and the treatment line that patients with presumptive TB may receive under the *SSM* strategy. Based on the observed TB strain (ds-TB or MDR-TB) and history of TB treatment, individuals may receive first-line regimen (rifampicin/isoniazid/pyrazinamide/ethambutol), retreatment regimen (rifampicin/isoniazid/pyrazinamide/ethambutol/streptomycin), or second-line regimen (kanamycin/levofloxacin/ethionamide/cycloserine/pyrazinamide/ethambutol) [14,47].

**Fig B. Diagnostic and treatment algorithm for *Xpert* strategy.**


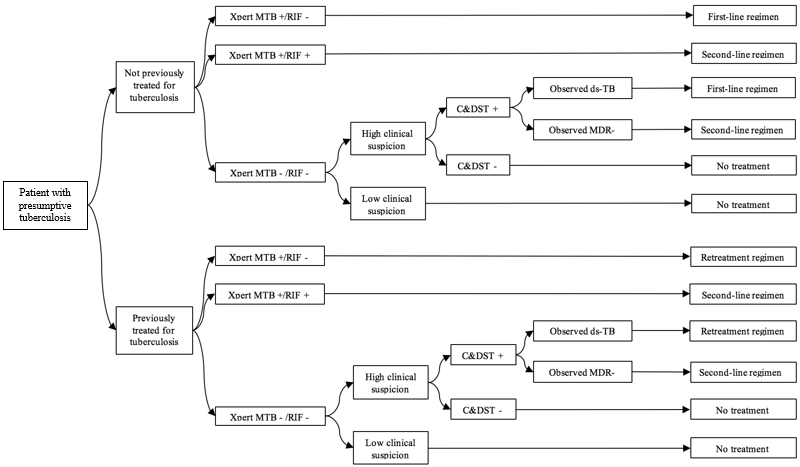


Abbreviations: MTB: *Mycobacterium tuberculosis*. RIF: rifampicin. C&DST: culture and drug-susceptibility testing. TB: tuberculosis.
ds-TB: drug-susceptible tuberculosis. MDR-TB: multidrug-resistant tuberculosis.

Schematic shows the possible combination of tests and the treatment line that patients with presumptive TB may receive under the *Xpert* strategy. Individuals with “high clinical suspicion” for TB, despite a negative Xpert result, may receive confirmatory C&DST. Based on the observed TB strain (ds-TB or MDR-TB) and history of TB treatment, individuals may receive first-line regimen (rifampicin/isoniazid/pyrazinamide/ethambutol),
retreatment regimen (rifampicin/isoniazid/pyrazinamide/ethambutol/streptomycin), or second-line regimen (kanamycin/levofloxacin/ethionamide/cycloserine/pyrazinamide/ethambutol) [14,47].

**Fig C. Diagnostic and treatment algorithm for *Truenat* strategies.**


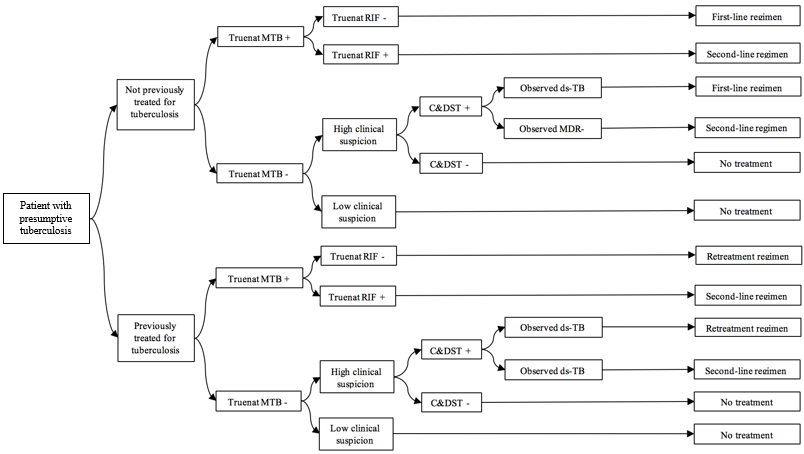


Abbreviations: MTB: *Mycobacterium tuberculosis*. RIF: rifampicin. C&DST: culture and drug-susceptibility testing. TB: tuberculosis.
ds-TB: drug-susceptible tuberculosis. MDR-TB: multidrug-resistant tuberculosis.

Schematic shows the possible combination of tests and the treatment line that patients with presumptive TB may receive under the *Truenat DMC* or *Truenat POC* strategy. Individuals undergo testing for RIF-resistance only if they first receive a positive test result for TB. Individuals with “high clinical suspicion” for TB, despite a negative Truenat result, may receive confirmatory C&DST. Based on the observed TB strain (ds-TB or MDR-TB) and history of TB treatment, individuals may receive first-line regimen (rifampicin/isoniazid/pyrazinamide/ethambutol), retreatment regimen (rifampicin/isoniazid/pyrazinamide/ethambutol/streptomycin), or second-line regimen (kanamycin/levofloxacin/ethionamide/cycloserine/pyrazinamide/ethambutol) [14,47].

**Fig D. Overview of TB states in the simulation model.**

**
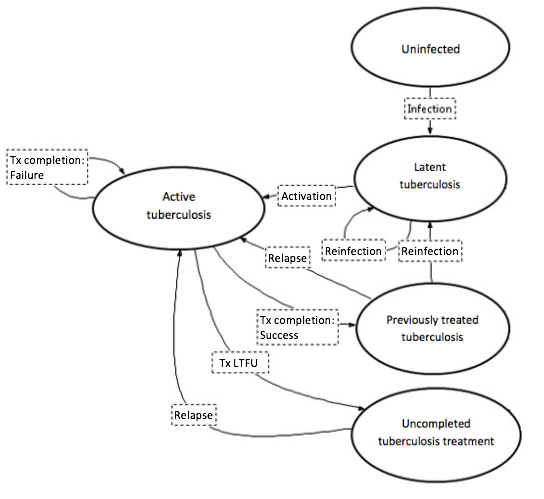
**

Abbreviation: Tx: treatment. LTFU: loss to follow-up. Displayed is a simplified diagram of the TB model (CEPAC model technical specifications are available at http://www.massgeneral.org/mpec/cepac/). The circles represent “states” of TB natural history, and the arrows represent the possible monthly transitions that may occur between states. Simulated individuals also may remain in their current state (arrows not depicted). Individuals can transition from any of these states to the “Dead” state (also not depicted for simplicity).

**Fig E.** **Incremental cost-effectiveness ratios of *Truenat POC* and *Xpert* over different time horizons.**

1. **Cost-effectiveness of *Truenat POC*, compared to *SSM* and *Xpert*, over different time horizons.**


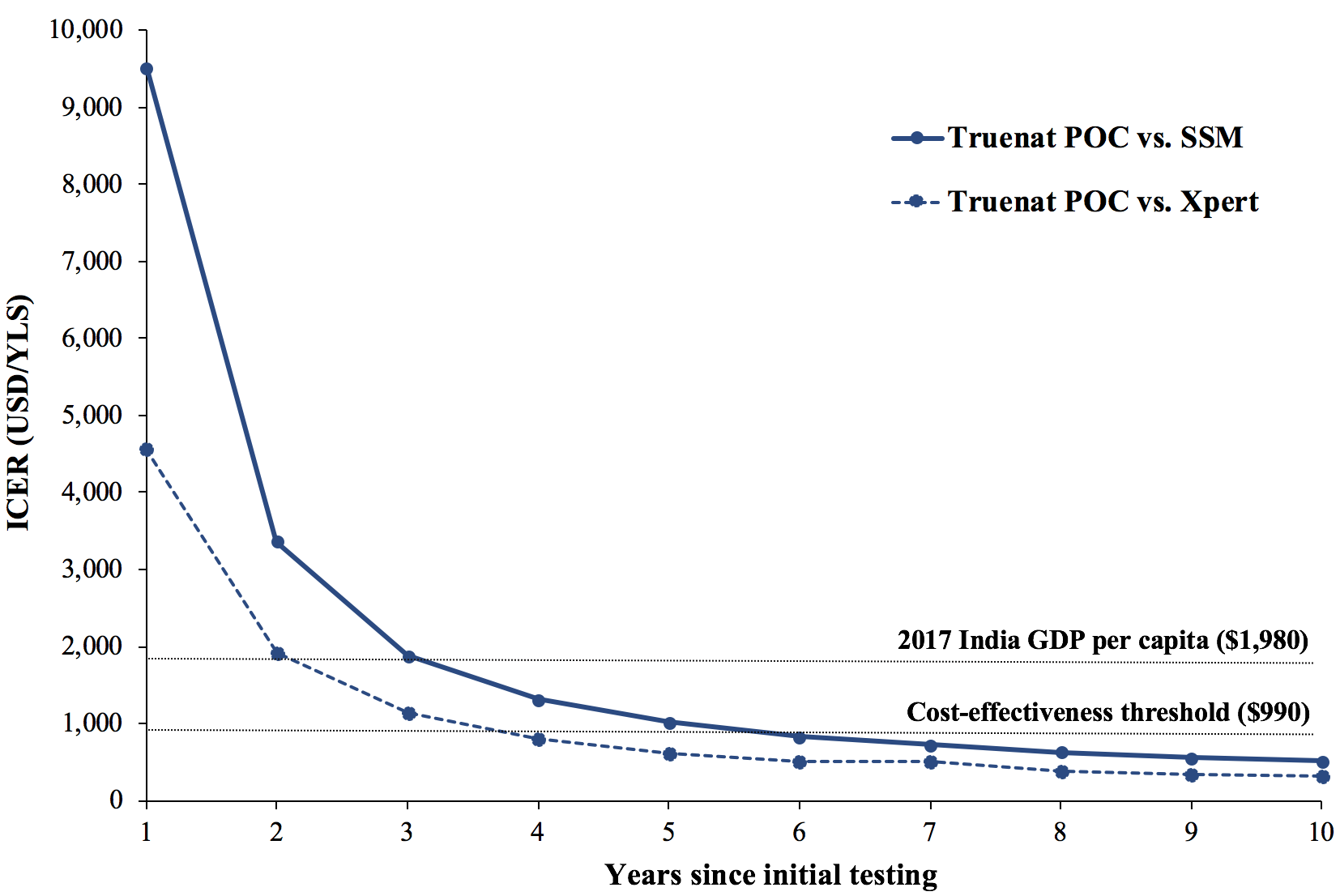


1. **Cost-effectiveness of *Xpert*, compared to *SSM*, over different time horizons.**

**
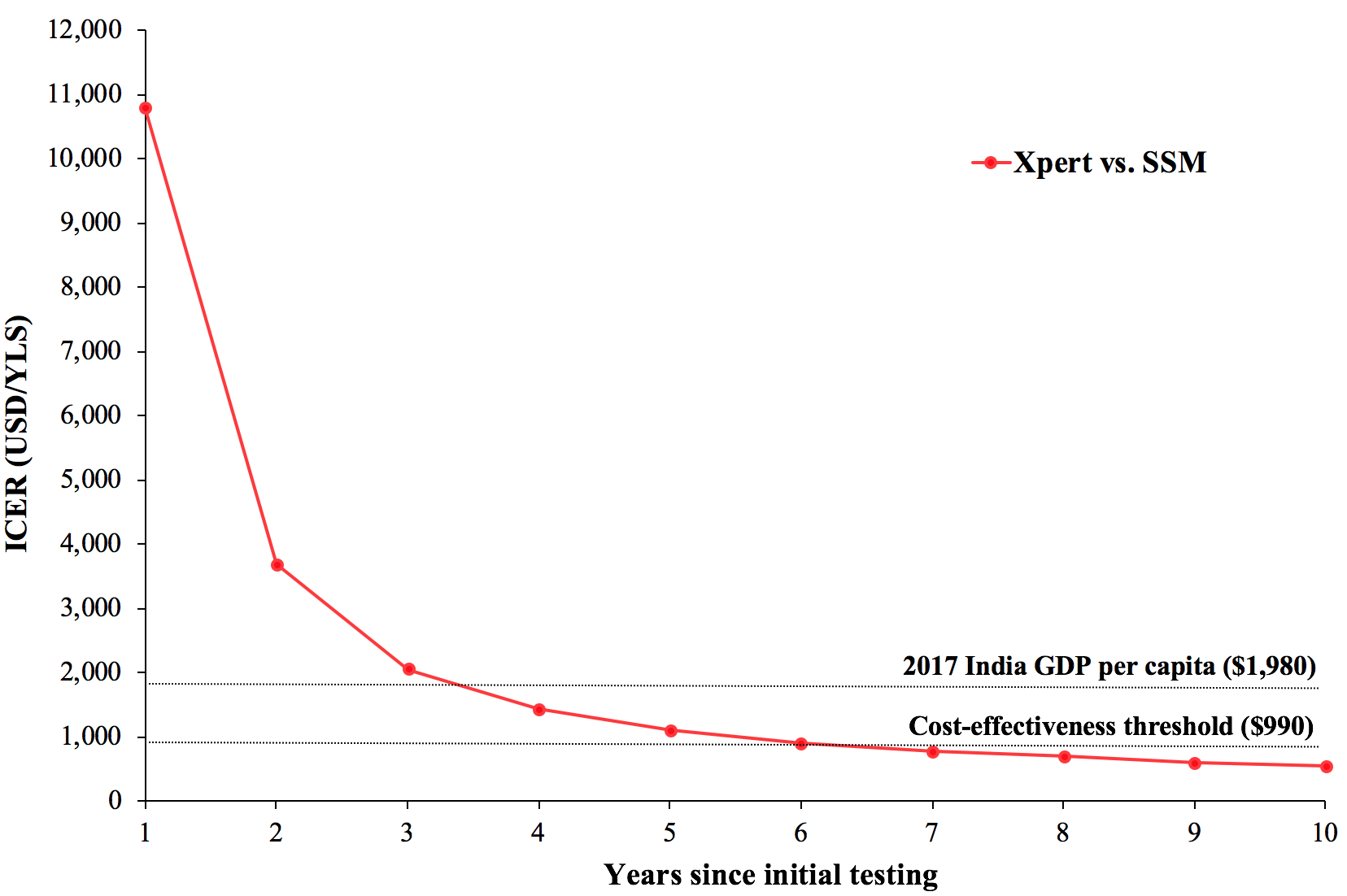
**

Abbreviations: POC: point of care. ICER: incremental cost-effectiveness ratio. YLS: year-of-life saved. GDP: gross domestic product. *SSM*: sputum smear microscopy strategy.

In the top graph (a), the blue solid line represents the ICERs of *Truenat POC*, compared to *SSM*, over different time horizons, and the blue dashed line represents the ICERs of *Truenat POC*, compared to *Xpert*, over different time horizons. In the bottom graph (b), the red solid line represents the ICERs of *Xpert*, compared to *SSM*, over different time horizons. In both graphs, the upper horizontal line represents the GDP *per capita* of India in 2017 ($1,980) [13]. The lower horizontal line represents the cost-effectiveness threshold, defined as 50% the GDP *per capita* of India in 2017 ($990). ICERs <$990/YLS (below lower horizontal line) are considered cost-effective.

**Fig F. Additional one-way sensitivity analyses of model parameters**.


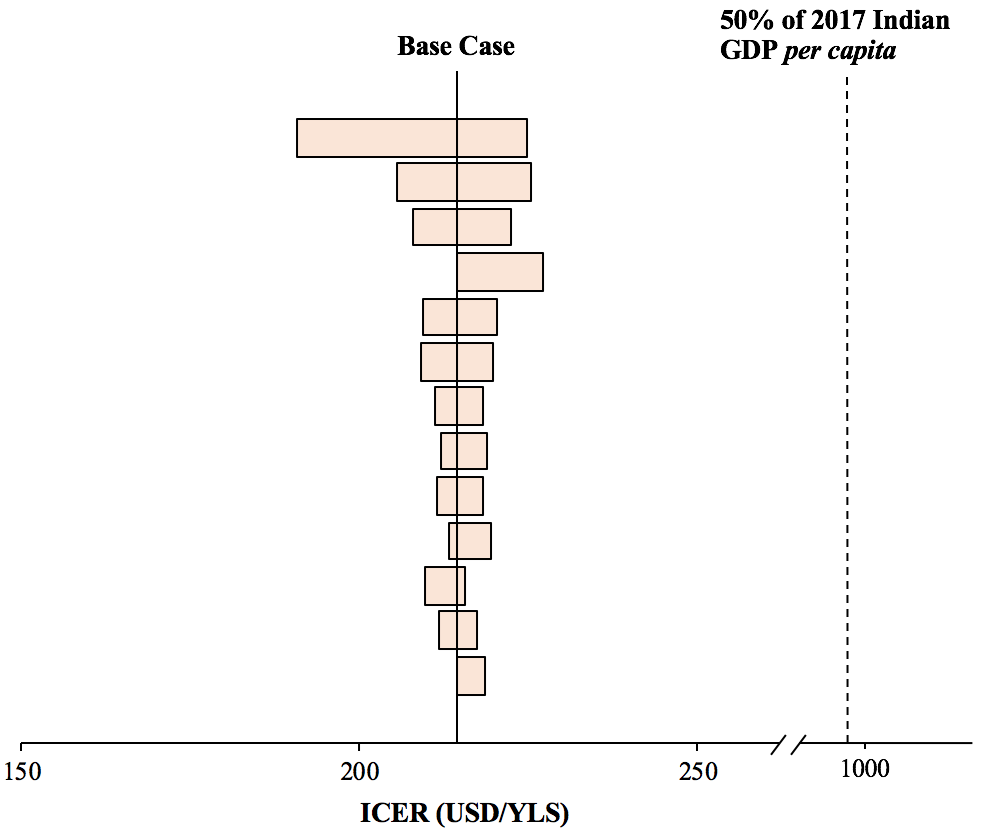


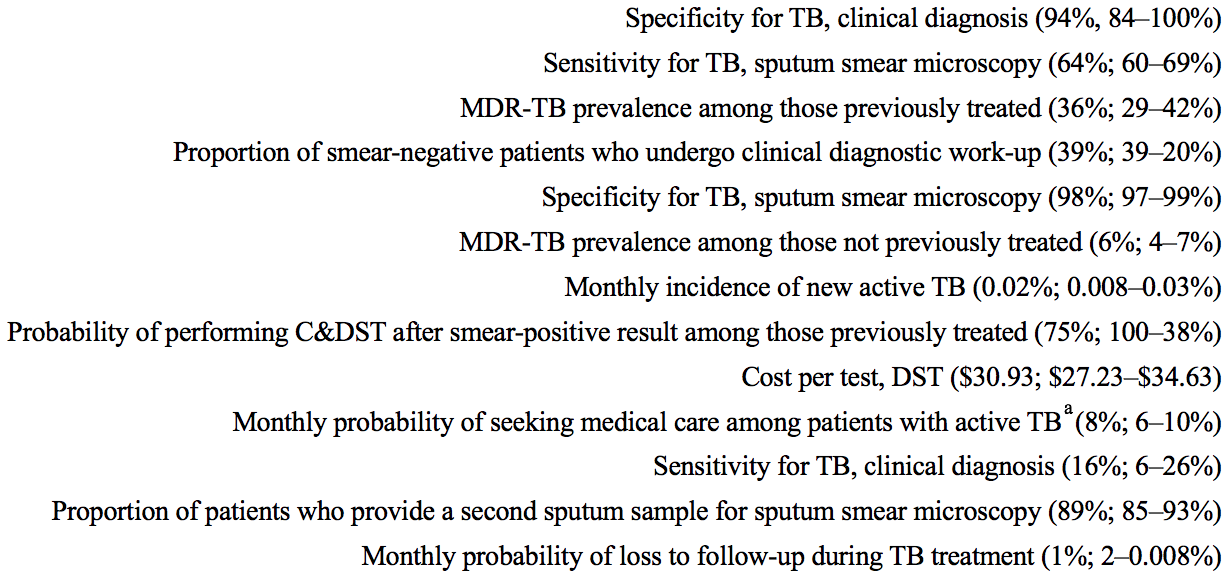


Abbreviations: TB: tuberculosis. MDR-TB: multidrug-resistant tuberculosis. “previously treated”: previously treated for TB.

C&DST: culture and drug-susceptibility test. DST: drug-susceptibility test. GDP: gross domestic product. ICER: incremental cost-effectiveness ratio.

USD: United States dollars. YLS: year-of-life saved.
One-way sensitivity analysis comparing the impact of model parameters on the ICER of Truenat used at point-of-care (*Truenat POC*) compared to sputum smear microscopy (*SSM*) strategy. Horizontal bars represent ranges of ICERs when varying each model parameter across different values. The vertical grey dashed line represents 50% of the gross domestic product (GDP) *per capita* of India in 2017 ($990), which we consider the cost-effectiveness threshold [6,12,13]. ICERs <$990/YLS (left of dashed line) are considered cost-effective.

^a^This parameter applies to individuals with active TB after initial TB testing.
